# New Cretaceous antlion-like lacewings promote a phylogenetic reappraisal of the extinct myrmeleontoid family Babinskaiidae

**DOI:** 10.1038/s41598-021-95946-z

**Published:** 2021-08-12

**Authors:** Xiumei Lu, Bo Wang, Xingyue Liu

**Affiliations:** 1 Institute of Ecological and Environmental Protection, Shanghai Academy of Agricultural Sciences, Shanghai, 201403 China; 2 State Key Laboratory of Palaeobiology and Stratigraphy, Nanjing Institute of Geology and Palaeontology and Center for Excellence in Life and Paleoenvironment, Chinese Academy of Sciences, 39 East Beijing Road, Nanjing, 210008 China; 3 Department of Entomology, China Agricultural University, Beijing, 100193 China

**Keywords:** Palaeontology, Taxonomy, Phylogenetics

## Abstract

Babinskaiidae is an extinct family of the lacewing superfamily Myrmeleontoidea, currently only recorded from the Cretaceous. The phylogenetic position of this family is elusive, with inconsistent inferences in previous studies. Here we report on three new genera and species of Babinskaiidae from the mid-Cretaceous Kachin amber of Myanmar, namely *Calobabinskaia xiai* gen. et sp. nov., *Stenobabinskaia punctata* gen. et sp. nov., and *Xiaobabinskaia lepidotricha* gen. et sp. nov. These new babinskaiids are featured by having specialized characters, such as the rich number of presectoral crossveins and the presence of scaly setae on forewing costal vein, which have not yet been found in this family. The exquisite preservation of the Kachin amber babinskaiids facilitate a reappraisal of the phylogenetic placement of this family based on adult morphological characters. Our result from the phylogenetic inference combining the data from fossil and extant myrmeleontoids recovered a monophyletic clade composed of Babinskaiidae and another extinct family Cratosmylidae, and further assigned this clade to be sister group to a clade including Nemopteridae, Palaeoleontidae, and Myrmeleontidae. Babinskaiidae appears to be a transitional lineage between Nymphidae and advanced myrmeleontoids, with ancient morphological diversification.

Babinskaiidae is an extinct lacewing family belonging to the superfamily Myrmeleontoidea, presently known with 13 species in nine genera^1^. The adults of Babinskaiidae are diagnosed by a combination of characters, including the filiform antennae, the presence of trichosors, the origin of RP + MA far distal to wing base, the presence of presectoral crossveins in both fore- and hind wings, and the reduction of hind wing A2 and A3 veins.

Babinskaiidae to date is only recorded from the Cretaceous, lasting a timespan of ca. 35 million years from the Barremian to the Cenomanian. Besides a single species from the Lower Cretaceous Zaza Formation of Russia, all the other babinskaiids known are found from the Lower Cretaceous Crato Formation of Brazil, and the mid-Cretaceous Kachin amber of northern Myanmar^2–11^, suggesting richer species diversity of this family from the Gondwanan landmasses. Notably, more than a half of known species of Babinskaiidae (seven species in six genera) come from the mid-Cretaceous Kachin amber^1^ (Table S1).

Despite definite myrmeleontoid affinity, the phylogenetic position of Babinskaiidae within Myrmeleontoidea is still elusive^7^. This family was originally established as a subfamily of Nymphidae (split-footed lacewings)^2^, and subsequently elevated to an independent family^12^. Martins-Neto^13,14^ postulated that Babinskaiidae is sister to a myrmeleontoid clade including Ascalaphidae + (Nemopteridae + (Araripeneuridae + (Palaeoleontidae + Myrmeleontidae))) in a phylogenetic analysis primarily based on fossil taxa of Myrmeleontoidea from the Lower Cretaceous of Brazil. Similar result was also obtained in Yang et al.^15^. However, Makarkin et al.^7^ divided Myrmeleontoidea into two epifamilies, i.e., Nymphidoidae and Myrmeleontoidae, and assigned Babinskaiidae together with Nymphidae into Nymphidoidae based on the presence of trichosors and the long MP that is not fused with CuA.

Here we describe three new species of Babinskaiidae, each representing a new genus, from the mid-Cretaceous of Myanmar, namely *Calobabinskaia xiai* gen. et sp. nov., *Stenobabinskaia punctata* gen. et sp. nov., and *Xiaobabinskaia lepidotricha* gen. et sp. nov. Remarkably, some specialized morphological characters are found in these new babinskaiids, such as the split arolium, the rich number of presectoral crossveins, and the presence of scaly setae on forewing, which highlight the morphological diversity of this extinct myrmeleontoid family. With the present finding, a broad spectrum of babinskaiid taxa is available for evaluating the phylogenetic position of this family. As such, a phylogenetic inference on the relationships among fossil and extant families of Myrmeleontoidea is performed based on adult morphological characters. Our results shed light on the early evolution of Myrmeleontoidea, an advanced clade of lacewings.

## Results

### Systematic palaeontology

Class Insecta Linnaeus, 1758

Order Neuroptera Linnaeus, 1758

Superfamily Myrmeleontoidea Latreille, 1802

**Family Babinskaiidae Martins-Neto and Vulcano, 1989**

Type genus: *Babinskaia* Martins-Neto and Vulcano, 1989

*Revised diagnosis* (1) Antenna filiform, longer than half of forewing length; (2) wings slenderly elongate, slightly broadened distad in most genera; (3) fore- and hind wing similar in shape and size, but hind wing sometimes strongly narrowed; (4) nygmata absent; (5) trichosors present along wing margin of both fore- and hind wings; (6) forewing 1r-m absent; (7) origin of RP + MA far distal to wing base, around midpoint of wing; (8) a long hypostigmal cell present; (9) several presectoral crossveins present in both fore- and hind wings; (10) MP single, pectinately branched in most genera; (11) forewing oblique vein absent between MP and CuA; (12) forewing CuA running parallel with posterior margin for a long distance; (13) forewing CuP fused with A1, or connected A1 by a short crossvein, or approximating A1 without any crossvein; (14) hind wing anal space very small, with reduced A3.

**Genus** ***Calobabinskaia*** **gen. nov.**

(Figs. 1, 2, 3)

**Figure 1:**
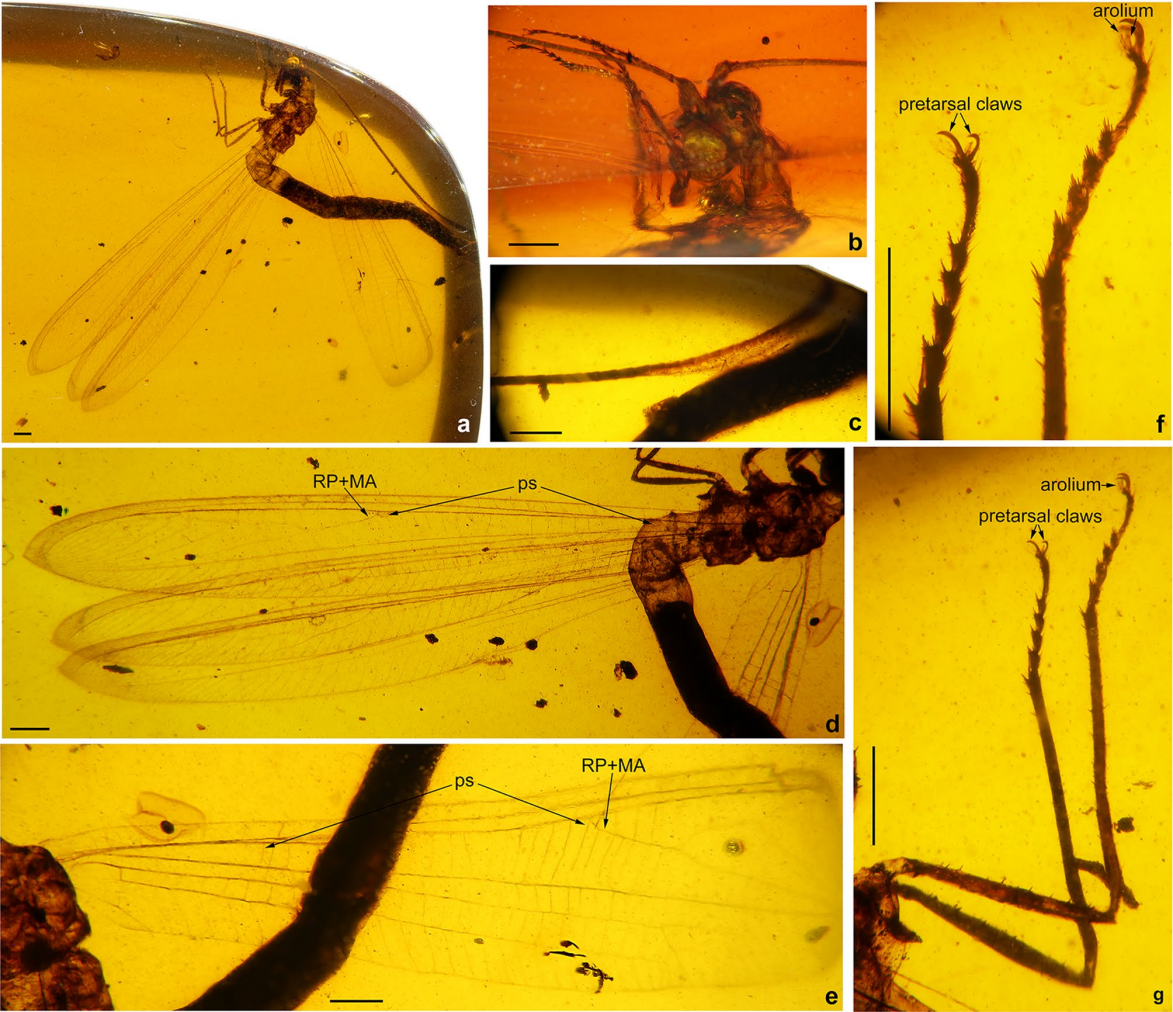
*Calobabinskaia xiai* sp. nov., holotype female. (**a**) habitus photograph, dorsolateral view; (**b**) photograph of head, dorsolateral view; (**c**) photograph of antennal apex; (**d**) photograph of left wings; (**e**) photograph of right forewing; (**f**) photograph of metatarsi; (**g**) photograph of hind legs. Scale bar = 1.0 mm.

**Figure 2:**
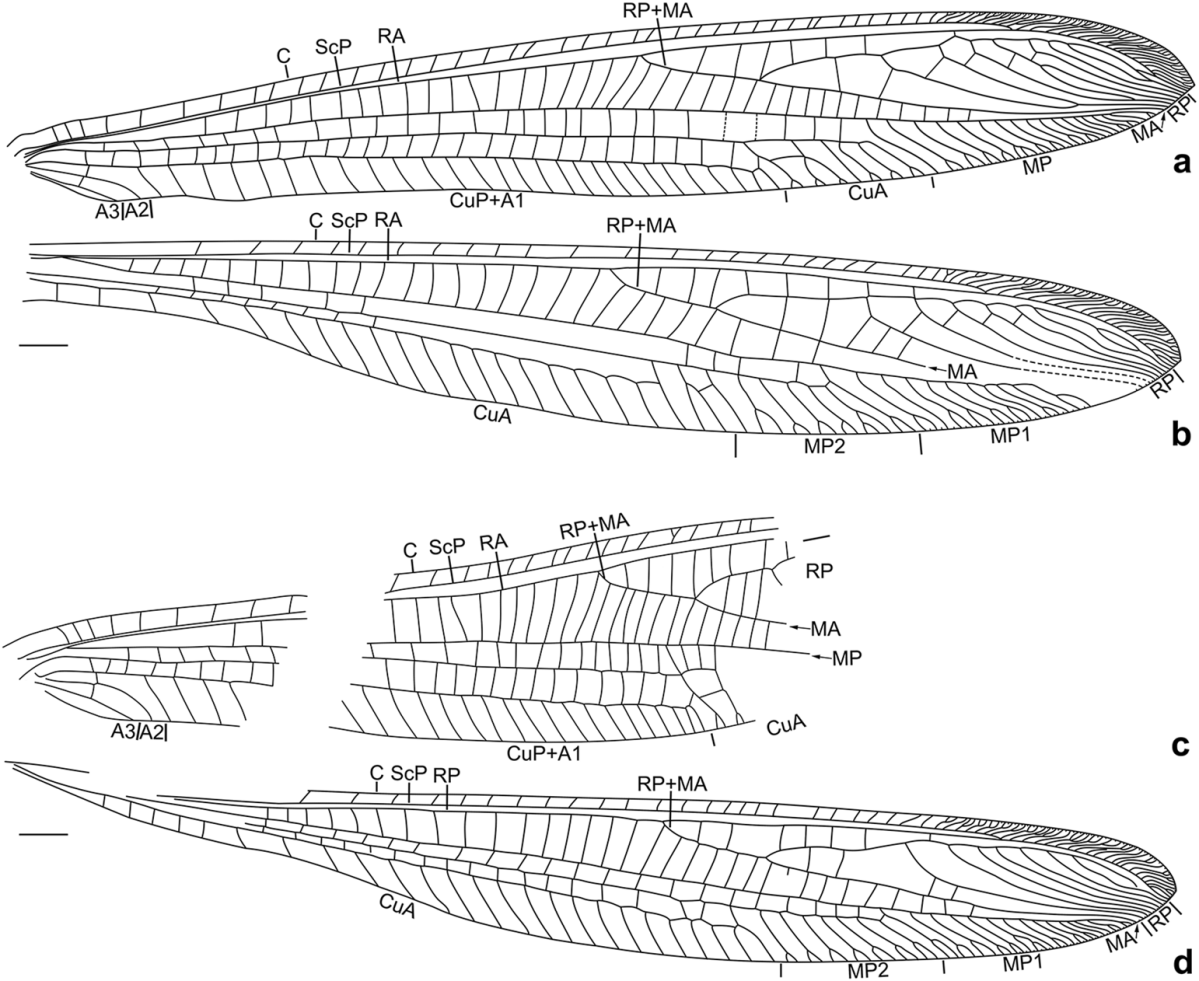
*Calobabinskaia xiai* sp. nov., holotype female. (**a**) drawing of left forewing (drawn by XML); (**b**) drawing of left hind wing (drawn by XML); (**c**) drawing of right forewing (drawn by XML); (**d**) drawing of right hind wing (drawn by XML). Scale bar = 1.0 mm.

**Figure 3:**
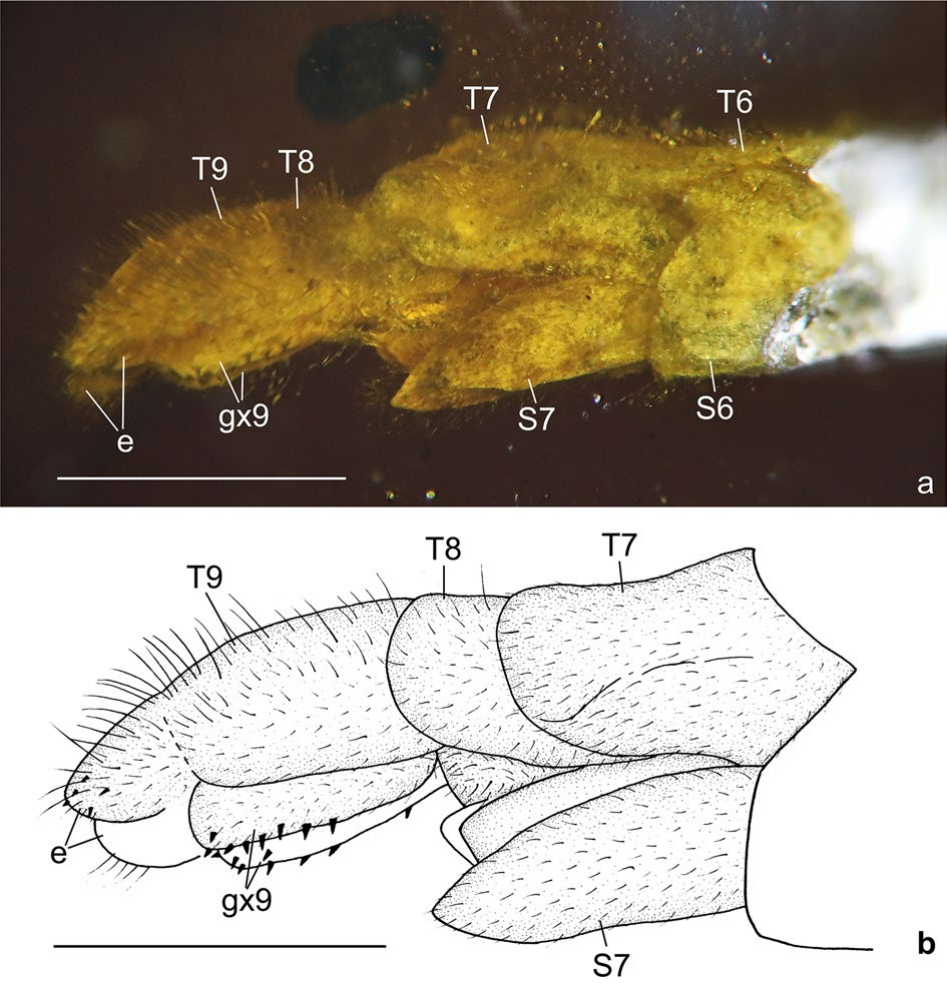
*Calobabinskaia xiai* sp. nov., holotype female. (**a**) photograph of genitalia, lateral view; (**b**) drawing of genitalia, lateral view (drawn by XML). Scale bar = 1.0 mm.

LSID: urn:lsid:zoobank.org:act:E84839F7-DFB7-4978-A835-16001DF964B0

Type species: *Calobabinskaia xiai* sp. nov

*Diagnosis* The new genus can be distinguished from the other genera of Babinskaiidae by a combination of the following characters: (1) large-sized babinskaiids, with forewing length ca. 22 mm [shared by *Gigantobabinskaia* Makarkin and Staniczek^10^ and *Stenobabinskaia* gen. nov., but slightly longer than these two genera; 9–15 mm in remaining genera]; (2) wings strongly narrowed, ca. 6.5 times as long as wide [shared by *Stenobabinskaia* gen. nov.; 4.5 times as long as wide in most genera]; (3) apex of antenna slightly dilated distally [antenna without dilated apex in the remaining genera with preserved antenna]; (4) forewing costal space strongly narrowed, almost as wide as subcostal space [shared by *Stenobabinskaia* gen. nov.; 2–3 times as wide as subcostal space in the other genera]; (5) numerous crossveins present in most part of wing except RP space in both wings [shared by *Stenobabinskaia* gen. nov.; crossveins much fewer in the other genera]; (6) 19 presectoral crossveins present in fore- and hind wings [10–11 in *Stenobabinskaia* gen. nov.; forewing with 4–7 presectoral crossveins and hind wing with 1–5 presectoral crossveins in most genera]; (7) RP + MA originating slightly distal to midpoint of wing in both wings [proximal to midpoint of wing in most genera; at midpoint of wing in *Neliana* Martins-Neto^12^ and *Pseudobabinskaia* Makarkin et al.^7^; distal to midpoint of wing in *Calobabinskaia* gen. nov.; unknown in *Burmobabinskaia* Lu et al.^6^]; (8) several RP branches fused with neighboring branches, especially in forewing [shared by *Stenobabinskaia* gen. nov.; absent in the other genera]; (9) one gradate series of crossveins present in forewing [shared by most babinskaiid genera; two or more series in *Stenobabinskaia* gen. nov. and *Xiaobabinskaia* gen. nov.]; (10) forewing CuP and hind wing CuA extremely long, terminating posterior to midpoint of hind margin [shared by *Stenobabinskaia* gen. nov.; relatively short, terminating at midpoint of hind margin in *Xiaobabinskaia* gen. nov.; much shorter, terminating proximal to midpoint of hind margin in most genera]; (11) forewing A1 proximally fused with CuP [shared by *Pseudoneliana* Huang et al.^9^, *Stenobabinskaia* gen. nov. and *Xiaobabinskaia* gen. nov.; proximally separated with each other in the other genera with preserved anal veins]; (12) arolium bilobed [simple or unknown in the other genera].

*Etymology* From “*Calo*-” (Greek, meaning “beautiful”) and “*babinskaia*” (the type genus-name of Babinskaiidae), in reference to the beautifully preserved material of this new genus with remarkable characters in Babinskaiidae. Gender: Feminine.

*Remarks* The new genus is placed in Babinskaiidae based on the presence of trichosors and multiple presectoral crossveins in both fore- and hind wings. The new genus is among the large-sized babinskaiids, together with *Gigantobabinskaia* and *Stenobabinskaia* gen. nov, with forewing length over 20 mm. However, it can be easily distinguished from *Gigantobabinskaia* by the narrowed wings, the presence of more than 15 presectoral crossveins, and the origin of RP + MA distal to midpoint of wing. The new genus is similar to *Stenobabinskaia* gen. nov. by the distinctly elongated wings, the presence of multiple presectoral crossveins, the bifurcated CuA branches in the forewing and the long CuP in the hind wing, but it differs from the latter genus by the fewer branches of RP and sparse crossveins in RP space, as well as the more distad origin of RP + MA.

***Calobabinskaia xiai*** **sp. nov.**

(Figs. 1, 2, 3)

LSID: urn:lsid:zoobank.org:act:1CD4D82D-DCCE-40BD-A56D-A9A32DA1B783

*Diagnosis* Same as for the genus.

*Description* Body length 25.63 mm; head 1.78 mm long, 0.76 mm wide; antenna length 19.11 mm; diameter of compound eye 0.81 mm; forewing 22.28 mm long, 3.40 mm wide; hind wing 20.74 mm long, 3.09 mm wide; abdomen length 18.52 mm.

Head with vertex medially domed; compound eyes large, semi-globular; antenna filiform, more than half of forewing length, slightly dilated distally; pedicel almost as long as but slightly narrower than scape.

Prothorax slender, longer than wide; meso- and metathorax robust. Wings strongly narrowed, ca. 6.5 times as long as wide, transparent and immaculate; single trichosor present between veins along distal margin in both fore- and hind wings.

Forewing: Costal space distinctly narrowed, almost as wide as subcostal space, but narrower than radial space, with 40 simple crossveins on proximal 4/5, and 26 crossveins and veinlets of ScP + RA on distal 1/5, which are mostly forked marginally; subcostal crossveins absent; 19 presectoral crossveins present; RP + MA originated from R slightly distal to midpoint of wing; RP pectinately branched from its proximal 1/3, with one rp-ma crossvein in left forewing and three in right forewing between stem of RP and MA; RP with eight branches, mostly simple, but RP4 deeply forked; RP1 fused with RP2 distally, RP3 fused with anterior branch of RP4 distally, RP7 fused with RP8 distally; one gradate series of four crossveins present near branching points of RP branches; MA simple; MP long and nearly straight, pectinately branched from distal 1/4, with 18 branches, mostly bearing a marginal fork; 27 crossveins present between MP and CuA; CuA and CuP diverging near wing base; CuA pectinately branched, with six branches, mostly bearing a marginal fork; CuP long, proximally fused with A1, terminating at same level of diverging point between RP and MA, with 28 pectinate, short and simple branches; 29 cua-cup crossveins present; A2 and A3 simple, connected with each other by a short a2-a3 crossvein.

Hind wing: Proximally distinctly narrowed, remaining part nearly as wide as forewing; costal space slightly wider than subcostal space, with at least 24 simple crossveins on proximal 4/5, and 25 crossveins and veinlets of ScP + RA on distal 1/5; subcostal crossveins absent; 19 presectoral crossveins present; RP + MA originating distal to midpoint of wing; two rp-ma crossveins present between stem of RP and MA; RP pectinately branched from its proximal 1/3 into eight simple branches, with distal-most two ones fused distally; one gradate series of four crossveins present near branching points of RP branches; MP1 and MP2 straight and long, both pectinately branched from distal 1/5, respectively with 15 and seven branches, mostly bearing a marginal fork; CuA long, terminating at same level of diverging point between MA and RP, with ca. 25 pectinate simple branches.

Legs slender, with sparse short setae; tarsus 5-segmented; tarsomeres 1–4 gradually shortened; tarsomere 5 longest, almost as long as combined length of tarsomeres 2–4; pretarsal claws curved, equal in length and shape, with a bilobed arolium.

Abdomen slender and elongate, gradually narrowed distally up to genitalia. Tergum 7 and sternum 7 broad, nearly equal in length, sternum 7 distinctly tapered posteriorly. Genitalia: Tergum 8 very short and narrower than tergum 9; no distinct gonocoxites 8 discernible; tergum 9 longer than tergum 8; a pair of narrow valvate gonocoxites 9 present, bearing a row of teeth along ventral margin; ectoprocts paired, small, with apex slightly tapered; callus cerci not discernible.

*Etymology* The new species is dedicated to Mr. Fangyuan Xia, who kindly offered the specimen (the holotype) of this new species for present study.

*Type material* Holotype: LPAM BA-NEU-002. Amber piece preserving a nearly complete female adult of *Calobabinskaia xiai* gen. et sp. nov. It is polished in the form of a flattened elliptical cabochon, clear and transparent, with length × width 53.47 × 37.55 mm, height 15.38 mm.

**Genus** ***Stenobabinskaia*** **gen. nov.**

(Figs. 4, 5, 6)

**Figure 4:**
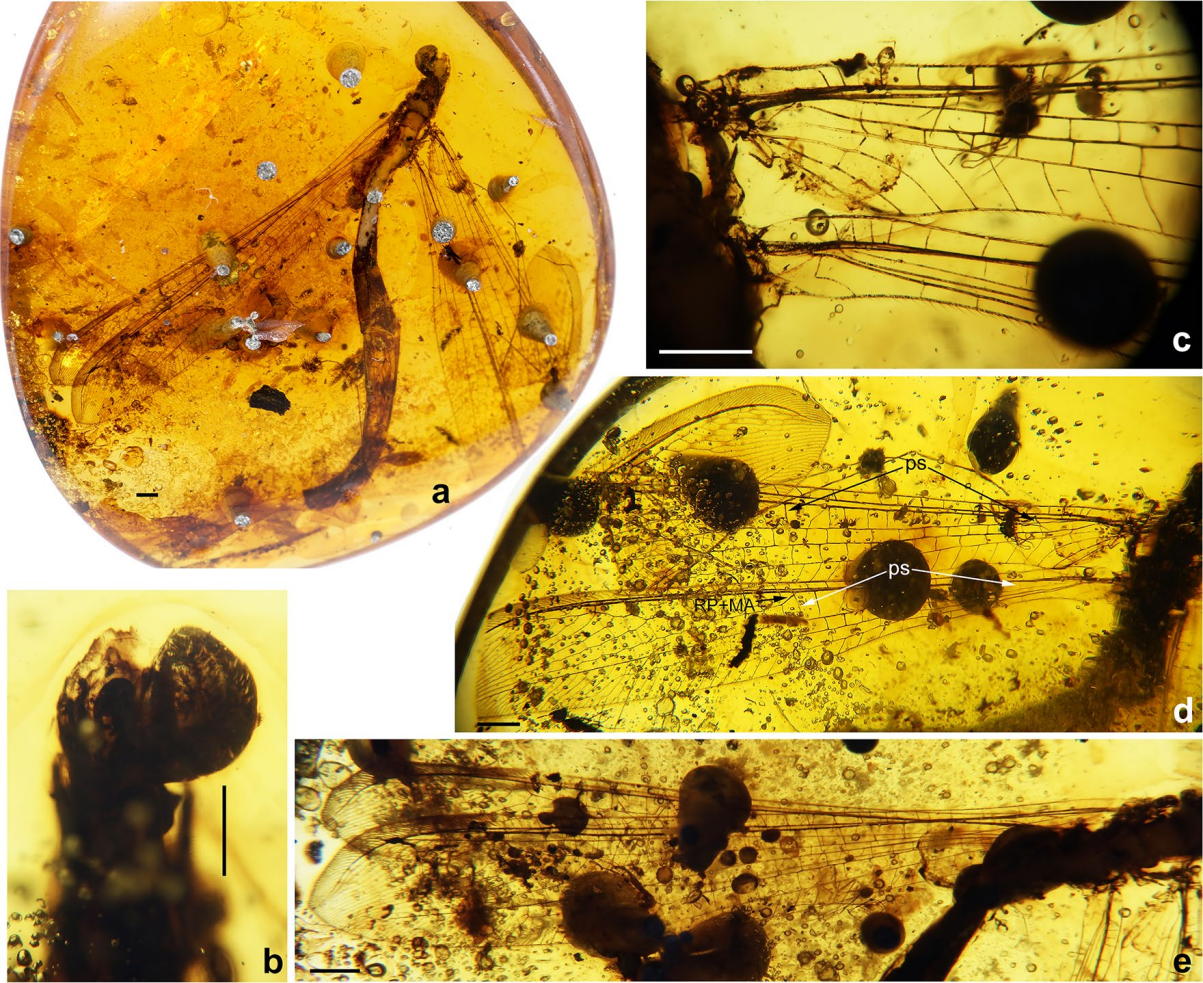
*Stenobabinskaia punctata* sp. nov., holotype male. (**a**) habitus photograph, dorsal view; (**b**) photograph of head and prothorax, dorsal view; (**c**) photograph of bases of right wings; (**d**) photograph of right wings; (**e**) photograph of left wings. Scale bar = 1.0 mm.

LSID: urn:lsid:zoobank.org:act:51AA6FFE-3BA6-4828-9E69-ACEC94A1A125

Type species: *Stenobabinskaia punctata* sp. nov.

*Diagnosis* The new genus can be distinguished from the other genera of Babinskaiidae by a combination of the following characters: (1) large-sized babinskaiids, with forewing length ca. 20 mm [shared by *Gigantobabinskaia* and *Calobabinskaia* gen. nov., but slightly shorter than these two genera; 9–15 mm in the other genera]; (2) wings strongly narrowed, ca. 6.5 times as long as wide [shared by *Calobabinskaia* gen. nov.; 4.5 times as long as wide in most genera]; (3) forewing costal space strongly narrowed, slightly wider than subcostal space [shared by *Calobabinskaia* gen. nov.; 2–3 times as wide as subcostal space in the other genera]; (4) crossveins densely spaced in both wings [shared by *Calobabinskaia* gen. nov.; much fewer in the other genera]; (5) at least 10–11 presectoral crossveins present in fore- and hind wings, with one of them distinctly sigmoid [19 in *Calobabinskaia* gen. nov.; 4–7 in most genera; no sigmoid presectoral crossvein in the other genera]; (6) RP + MA originating slightly proximal to midpoint of wing [shared by most genera; at midpoint of wing in *Neliana* and *Pseudobabinskaia*; distal to midpoint of wing in *Calobabinskaia* gen. nov.; unknown in *Burmobabinskaia*]; (7) several RP branches fused with neighboring branches, especially in forewing [shared by *Calobabinskaia* gen. nov.; absent in the other genera]; (8) forewing with three gradate series of crossveins [one gradate series present in most genera; more than three gradate series present in *Xiaobabinskaia* gen. nov.]; (9) forewing CuP and hind wing CuA extremely long, terminating posterial to midpoint of hind margin [shared by *Calobabinskaia* gen. nov.; relatively short, terminating at midpoint of hind margin in *Xiaobabinskaia* gen. nov.; much shorter, terminating proximad midpoint of hind margin in most genera]; (10) forewing A1 fused with CuP [shared by *Pseudoneliana*, *Calobabinskaia* gen. nov. and *Xiaobabinskaia* gen. nov.; separated from each other in the other genera with preserved anal veins].

*Etymology* From “*Steno*-” (Greek, meaning “narrow”) and “*babinskaia*” (the type genus-name of Babinskaiidae) in reference to the narrowly elongate wings in the new genus. Gender: Feminine.

*Remarks* The new genus resembles *Calobabinskaia* gen. nov., and the morphological comparison can be seen in the Remarks section of *Calobabinskaia* gen. nov.

***Stenobabinskaia punctata*** **sp. nov.**

(Figs. 4, 5, 6)

**Figure 5:**
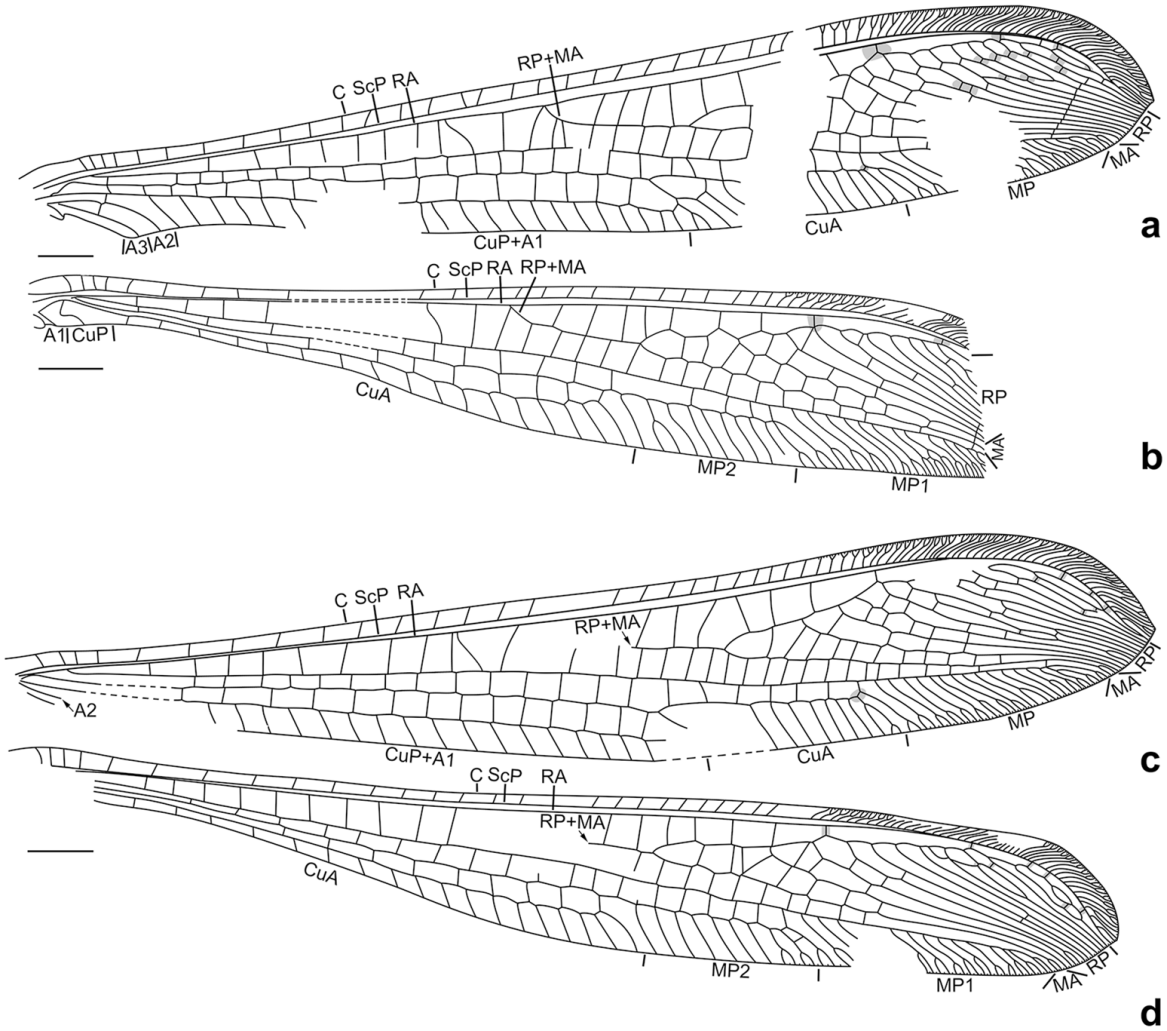
*Stenobabinskaia punctata* sp. nov., holotype male. (**a**) drawing of left forewing (drawn by XML); (**b**) drawing of left hind wing (drawn by XML); (**c**) drawing of right forewing (drawn by XML); (**d**) drawing of right hind wing (drawn by XML). Scale bar = 1.0 mm.

**Figure 6:**
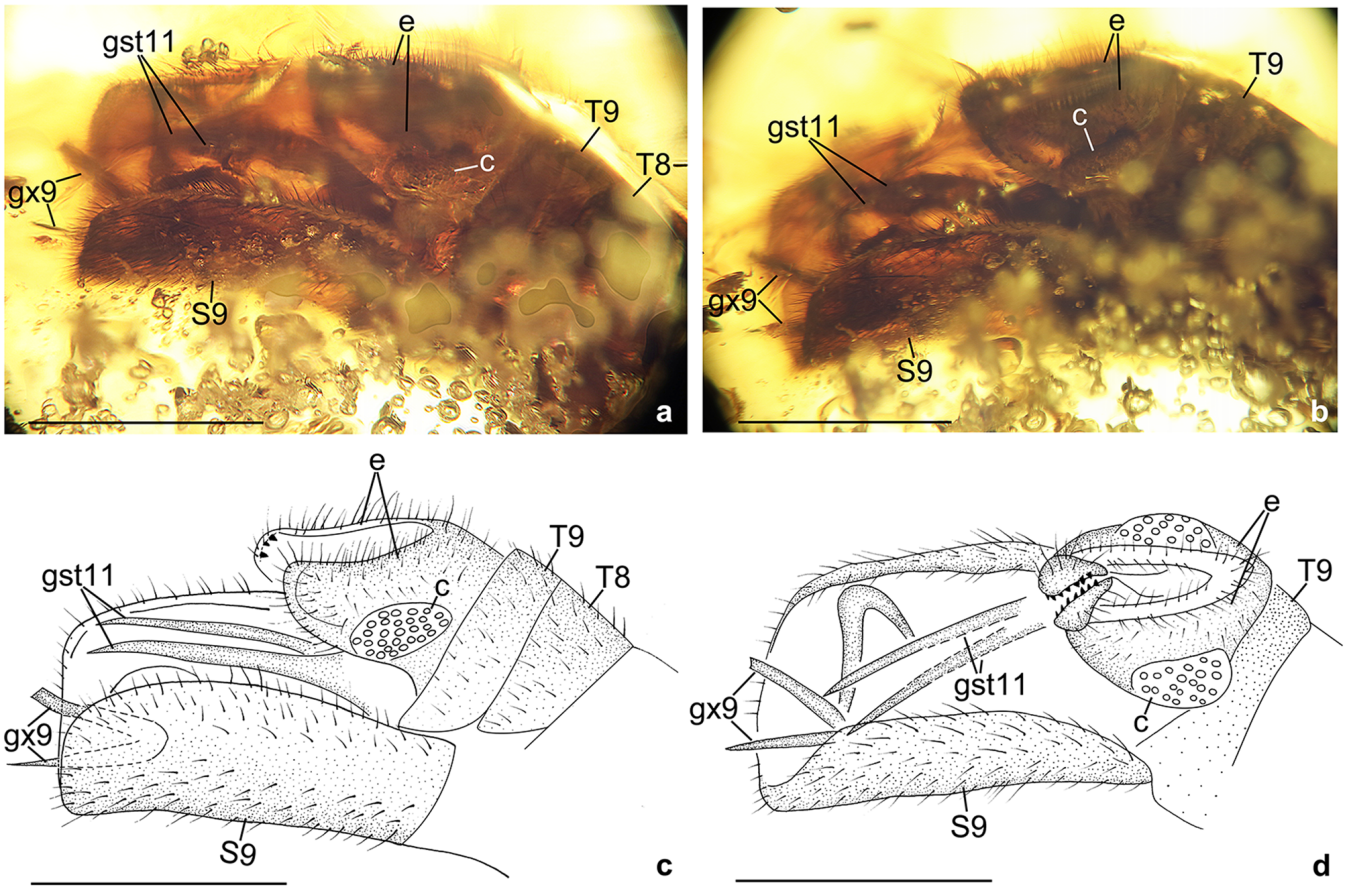
*Stenobabinskaia punctata* sp. nov., holotype male. **(a**) photograph of genitalia, lateral view; (**b**) photograph of genitalia, dorsal view; (**c**) drawing of genitalia, lateral view (drawn by XML); (**d**) drawing of genitalia, dorsal view (drawn by XML). Scale bar = 1.0 mm.

LSID: urn:lsid:zoobank.org:act:8235C299-9B3B-4319-A826-30FCFC1F5A89

*Diagnosis* Same as for the genus, besides dark spots present at distal 1/3 of both fore- and hind wings, mostly on crossveins between RP branches.

*Description* Male. Body length 23.09 mm; head 1.34 mm long, 1.84 mm wide; diameter of compound eye 1.17 mm; forewing 20.07 mm long, 3.11 mm wide; hind wing 18.41 mm long, 2.72 mm wide; abdomen length 17.91 mm.

Head with vertex medially domed; compound eyes large, semi-globular; antenna not preserved.

Prothorax much narrower than head, slightly wider than long; meso- and metathorax slightly wider than prothorax. Wings narrowly elongate, ca. 6.5 times as long as wide; wing spots present on apex of both fore- and hind wings, mostly on some crossveins of branching region of RP; single trichosor present between veins along distal margin in both fore- and hind wings.

Forewing: Costal space strongly narrowed, slightly wider than subcostal space, but narrower than radial space, with ca. 30 simple crossveins on proximal 3/4, and ca. 45 crossveins and veinlets of ScP + RA on distal 1/4, mostly forked marginally; subcostal crossveins absent; 11 presectoral crossveins present, one of them sigmoid and acutely angled with R; RP + MA originated from R slightly proximal to midpoint of wing; RP pectinately branched from its proximal 1/5, with one rp-ma crossvein between stem of RP and MA; RP with 13 branches, mostly simple, but RP5 and RP6 deeply forked; RP8 and RP9, RP12 and RP13 fused distally; at least four gradate series of crossveins present among branches of RP and MA at proximal half; an additional distal gradate series of crossveins present; MA only with a marginal fork; MP long and nearly straight, pectinately branched from distal 1/4, with 17 branches, mostly bearing a marginal fork; 22 crossveins present between MP and CuA; CuA and CuP diverging near wing base; CuA pectinately branched, with at least 10 branches, most of which are simple; CuP long, proximally fused with A1, terminating at same level of diverging point between RP and MA, pectinately branched with at least 23 short and simple branches; 18 cua-cup crossveins present; A2 and A3 simple, connected with each other by a short a2-a3 crossvein.

Hind wing: Proximally distinctly narrowed, remaining part nearly as wide as forewing; costal space narrow, nearly twice as wide as subcostal space, with at least 28 crossveins on proximal 3/4, and 45 crossveins and veinlets of ScP + RA on distal 1/4, mostly forked marginally; subcostal crossveins absent; at least 10 presectoral crossveins present, one of them sigmoid and acutely angled with R; RP + MA originating slightly proximal to midpoint of wing; RP pectinately branched from its proximal 1/5 into 13 mostly simple branches, with RP3 bearing a marginal fork; two rp-ma crossveins present in left forewing and three in right wing between stem of RP and MA; RP7 touched with RP8 at a point basally; RP8 and RP9 fused into a loop; RP12 and RP13 fused distally, then fused with R11 distally; at least two gradate series of crossveins present among branches of RP and MA at proximal half; an additional distal gradate series of crossveins present; MP1 and MP2 diverging near wing base, straight and long, both pectinately branched from its distal 1/4; MP1 with 15 branches, mostly marginally forked; MP2 with nine branches, mostly simple; CuA and CuP diverging near wing base; CuA long, terminating at same level of branching point between RP and MA, pectinately branched with 17 simple branches; CuP strongly sigmoid proximally, simple; A1 short and simple.

Legs not preserved.

Abdomen slenderly elongate, with segments 4–5 slightly widened. Male genitalia: Tergum 9 slightly shorter than tergum 8; sternum 9 large, about twice as long as tergum 9 plus ectoproct, straightly extending posteriad; putative gonocoxite 9 present, but probably detached from its original position, strongly curved medially with acute tip; putative gonostyli 11 present as a pair of long spinous sclerites, which are slightly shorter than sternum 9; ectoprocts paired, subtrapezoidal in lateral view, with large ovoid callus cerci, and distally with some short teeth.

*Etymology* The specific epithet “*punctata*” refers to the occurrence of wing spots in the new species.

*Type material* Holotype: NIGP176251. Amber piece preserving a nearly complete male adult of *Stenobabinskaia punctata* gen. et sp. nov. It is polished in the form of a flattened triangular cabochon, clear and transparent, with lengths of three sides 29.65 × 28.71 × 26.23 mm, height 7.07 mm.

**Genus** ***Xiaobabinskaia*** **gen. nov.**

(Figs. 7, 8, 9)

**Figure 7:**
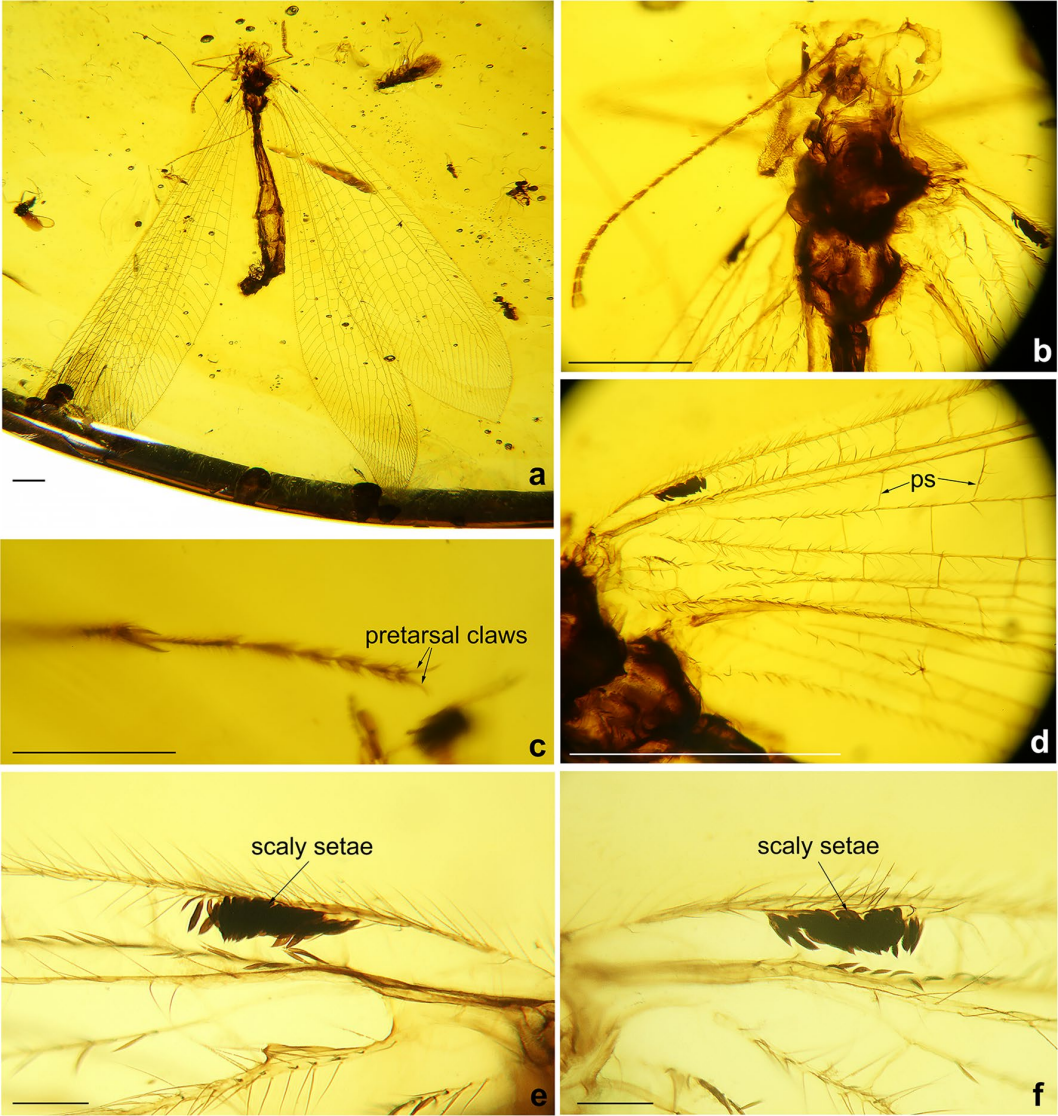
*Xiaobabinskaia lepidotricha* sp. nov., holotype female. (**a**) habitus photograph, dorsal view; (**b**) photograph of head and prothorax, dorsal view; (**c**) photograph of tarsus; (**d**) photograph of bases of right wings; (**e**) photograph of costal scaly setae in left forewing; (**f**) photograph of costal scaly setae in right forewing. Scale bar = 1.0 mm.

**Figure 8:**
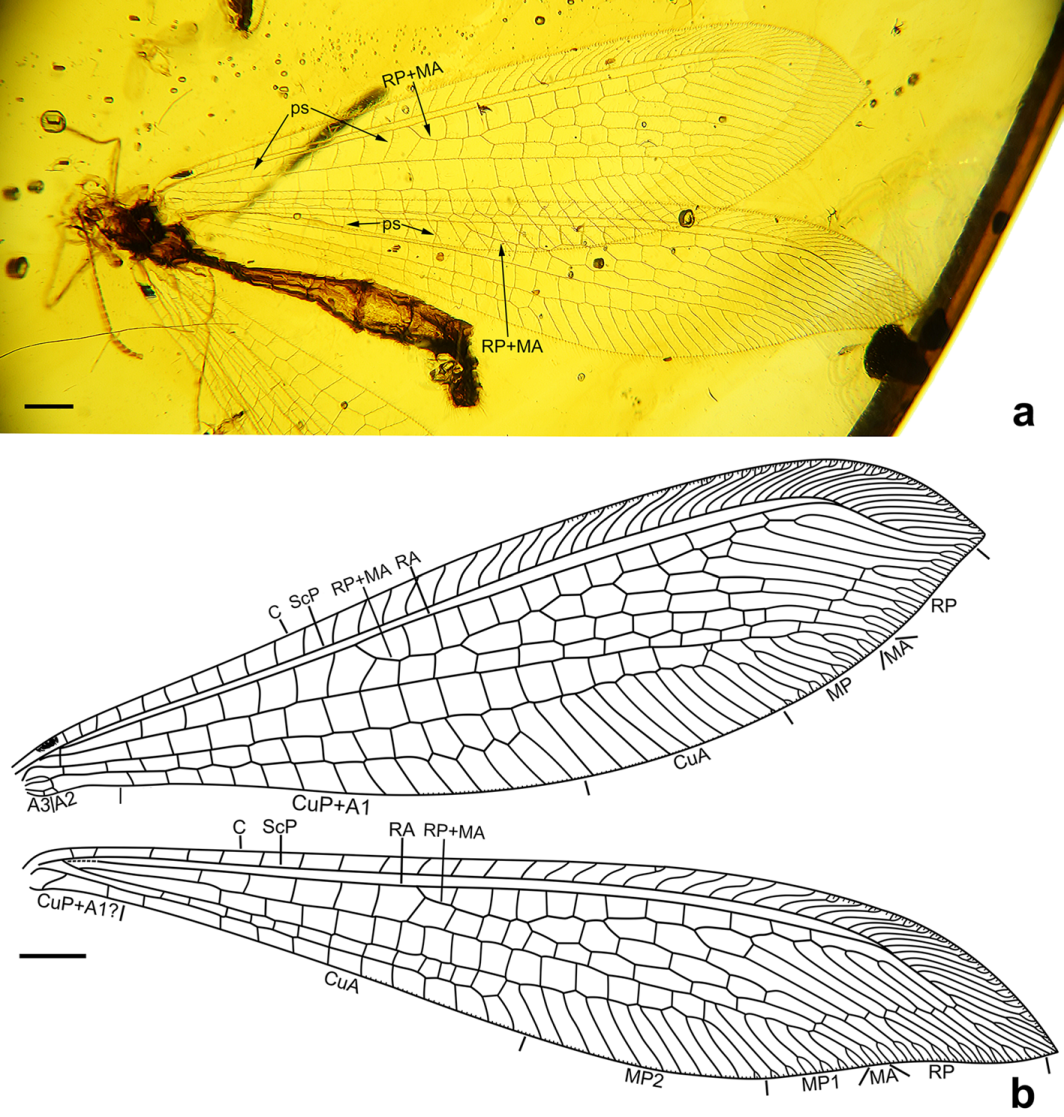
*Xiaobabinskaia lepidotricha* sp. nov., holotype female. (**a**) photograph of right wings; (**b**) drawing of right wings (drawn by XML). Scale bar = 1.0 mm.

**Figure 9:**
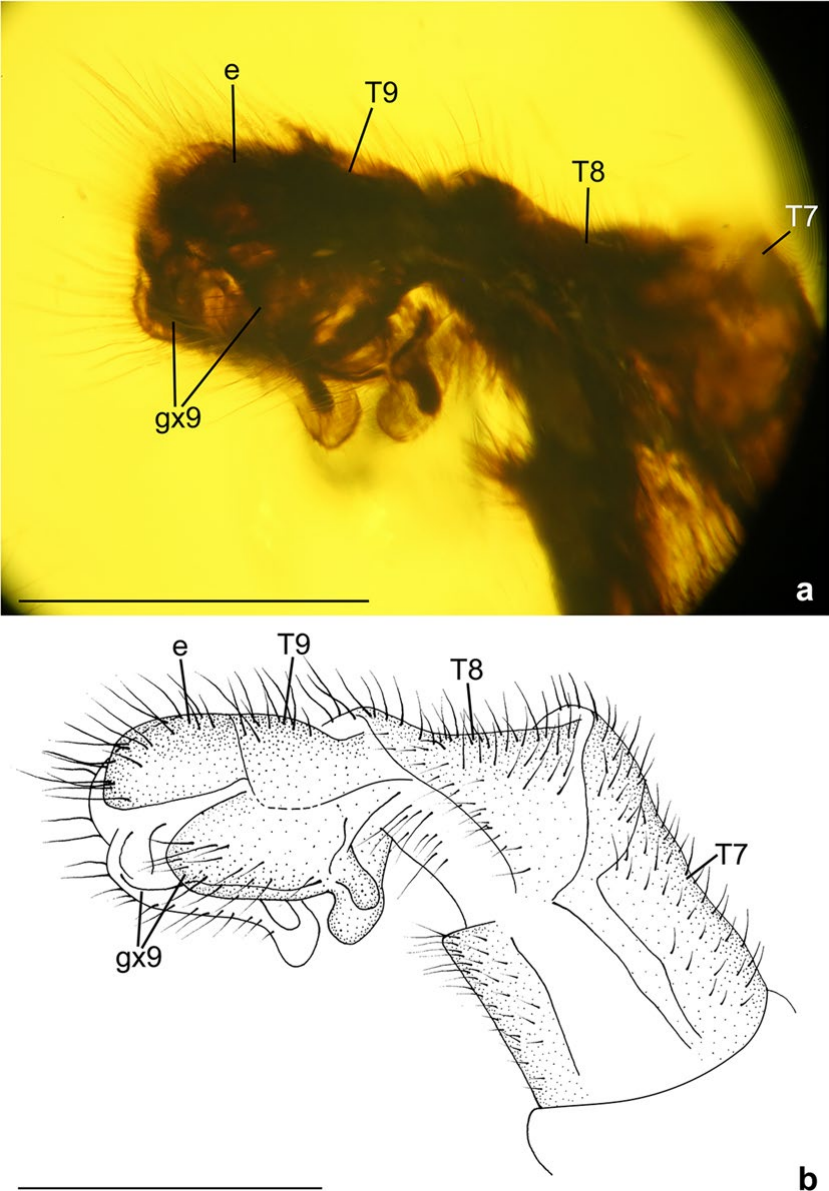
*Xiaobabinskaia lepidotricha* sp. nov., holotype female. (**a**) photograph of genitalia, lateral view; (**b**) drawing of genitalia, lateral view (drawn by XML). Scale bar = 1.0 mm.

LSID: urn:lsid:zoobank.org:act:0D29E44C-7C2D-47DD-A070-657F25B81ACE

Type species: *Xiaobabinskaia lepidotricha* sp. nov.

*Diagnosis* The new genus can be distinguished from the other genera of Babinskaiidae by a combination of the following characters: (1) medium-sized babinskaiids, with forewing length ca. 15 mm [ca. 10–13 mm in most genera of Babinskaiidae; more than 20 mm in *Gigantobabinskaia*, *Calobabinskaia* gen. nov. and *Stenobabinskaia* gen. nov.]; (2) a row of scaly setae present on proximal section of forewing costal vein, and a number of thick setae present along proximal sections of forewing ScP, MP, and A1 [no specialized setae on forewing veins in the other Kachin amber babinskaiids; unknown in genera preserved as compression fossils]; (3) hind wing with apex acutely tapered and slightly curved posteriad [shared by *Electrobabinskaia* Lu et al.^6^; feebly tapered in the other genera]; (4) six presectoral crossveins present in forewing, and at least five presectoral crossveins present in hind wing [forewing with 4–7 presectoral crossveins and hind wing with 1–5 presectoral crossveins in most genera; more than 10 presectoral crossveins present in *Calobabinskaia* gen. nov. and *Stenobabinskaia* gen. nov.]; (5) RP + MA originating at proximal 1/3 of wing in both fore- and hind wings [shared by *Electrobabinskaia* and *Gigantobabinskaia*; slightly shifted distad although still proximal to midpoint of wing in most genera; at midpoint of wing in *Neliana* and *Pseudobabinskaia*; distal to midpoint of wing in *Calobabinskaia* gen. nov.; unknown in *Burmobabinskaia*]; (6) RP with eight branches in both fore- and hind wings [shared by *Calobabinskaia* gen. nov. and *Electrobabinskaia*; 3–5 RP branches in most genera; more than 10 RP branches in *Gigantobabinskaia* and *Stenobabinskaia* gen. nov.]; (7) forewing with 15 or more crossveins among branches of RP and MA on proximal half [shared by *Stenobabinskaia* gen. nov.; forewing with 10 or less corresponding crossveins in most genera]; (8) a distal gradate series of crossveins present in both fore- and hind wings [shared by *Baisonelia* Ponomarenko^4^, *Parababinskaia* Makarkin et al.^7^, *Electrobabinskaia*, *Gigantobabinskaia*, *Stenobabinskaia* gen. nov.; absent in *Babinskaia* Martins-Neto and Vulcano^2^, *Calobabinskaia* gen. nov., *Neliana* and *Pseudobabinskaia*; unknown in the other genera]; (9) forewing CuP and hind wing CuA long, terminating at midpoint of hind margin [extremely long, terminating posterior to midpoint of hind margin in *Calobabinskaia* gen. nov. and *Stenobabinskaia* gen. nov.; much shorter, terminating proximad midpoint of hind margin in most genera]; (10) forewing A1 proximally fused with CuP, A2 approximating stem of A1 and distally abruptly curved posteriad [similar to *Pseudobabinskaia*, but A1 not fused with CuP; A2 and/or A3 relatively long and not curved posteriad in the other genera with CuP and A1 fused].

*Etymology* From “*Xiao*-” and “*Babinskaia*” (the type genus-group name of Babinskaiidae) in reference to Mrs. Xiao Jia, who kindly offered the specimen (the holotype) of the genus type for our study. Gender: Feminine.

*Remarks* The new genus resembles *Electrobabinskaia* and *Gigantobabinskaia* by the presence of distal gradate series of crossveins in both fore- and hind wings and the apex acutely tapered and curved posteriad in hind wing. However, it differs from the latter two genera by the presence of a row of scaly setae on proximal section of the forewing costal vein, the dense crossveins among branches of RP + MA, and the forewing A1 proximally fused with CuP. Besides, the new genus differs from *Electrobabinskaia* by the larger body size, the simple CuA branches, and the elongate CuP in the forewing, and it differs from *Gigantobabinskaia* by the smaller body size and the sparser RP branches.

***Xiaobabinskaia lepidotricha*** **sp. nov.**

(Figs. 7, 8, 9)

LSID: urn:lsid:zoobank.org:act:AD9E972D-9EBA-4C9A-8C63-DC017B75C994

*Diagnosis* Same as for the genus.

*Description* Female. Body length 10.76 mm; head 0.67 mm long, 1.51 mm wide; incomplate antenna length 3.40 mm; diameter of compound eye 0.68 mm; forewing 15.48 mm long, 4.19 mm wide; hind wing 15.92 mm long, 3.31 mm wide; abdomen length 10.00 mm.

Head with vertex medially domed; compound eyes large, semi-globular; antenna partly preserved; scape much wider and slightly longer than pedicel; flagellum with 22 flagellomeres preserved, each flagellomere distinctly shorter than and almost as wide as scape.

Prothorax narrower than head, slightly longer than wide; meso- and metathorax robust, much wider than prothorax. Wings broad, ca. 3.5 times as long as wide, transparent and immaculate; single trichosor between veins along distal margin but 3–5 trichosors present between distal costal crossveins and branches of CuA and CuP in forewing, or branches of MP2 and CuA in hind wing.

Forewing: Wing base strongly narrowed, with a row of scaly setae on proximal section of costal vein, and a number of thick setae along proximal sections of ScP, MP, and A1; costal space gradually widened distally, nearly three times as wide as subcostal space, distally distinctly wider than radial space, with ca. 27 simple crossveins on proximal 2/3, and 21 crossveins and veinlets of ScP + RA on distal 1/3, mostly marginally forked; subcostal crossveins absent; six presectoral crossveins present; RP + MA originating at proximal 1/3 of wing; RP pectinately branched from its proximal 1/5, with one rp-ma crossvein between stem of RP and MA; RP with eight branches, mostly bearing a marginal fork; 16 crossveins present among branches of RP and MA on proximal half; a distal gradate series of crossveins present; MA slightly zig-zagged, with a marginal fork; MP long and straight, branched from distal 1/6, with six branches, mostly deeply forked; 17 crossveins present between MP and CuA; CuA slightly zig-zagged, pectinately branched at distal 1/3, with 10 simple branches; CuP and A1 proximally fused, distally zig-zagged, terminating slightly distal to diverging point between RP and MA, pectinately branched into 14 simple branches; A2 approximating stem of A1, distally abruptly curved posteriad; A3 simple, with one a2-a3 crossvein.

Hind wing: Slightly longer and narrower than forewing, with base distinctly narrowed, and with apex acutely pointed and slightly bended posteriad; costal space nearly twice as wide as subcostal space, with 22 mostly simple crossveins on proximal 3/4 and 16 crossveins and veinlets of ScP + RA on distal 1/4, mostly forked marginally; subcostal crossveins absent; five presectoral crossveins present; RP + MA originating at proximal 1/3 of wing; RP pectinately branched from its proximal 1/5 into eight branches, mostly bearing a marginal fork; one rp-ma crossvein present between stem of RP and MA; eight crossveins present among branches of RP and MA on proximal half; a distal gradate series of crossveins present, with 10 corssveins; MP1 and MP2 diverging near wing base, long and slightly zig-zagged; MP1 pectinately branched distally, with six branches, each bearing a marginal fork; MP2 pectinately branched from distal 1/3, with 11 branches, most of which are simple except distal-most one bearing a small marginal fork; CuA long, with 11 simple branches, terminating at same level of diverging point between RP and MA; CuP and A1 possibly fused with CuA.

Legs slender, with sparse short setae; a pair of tibial spurs present; tarsus 5-segmented; tarsomere 1 longest, nearly twice as long as tarsomere 2; tarsomeres 3–5 shortest, nearly half length of tarsomere 2; each tarsomere slightly widened distally; pretarsal claws slender and slightly curved, equal in length and shape; arolium not discernible.

Abdomen slenderly elongate, with segments 4–6 much broader. Female genitalia: Tergum 8 nearly as long as tergum 9 plus ectoproct; ventral sclerites of segment 8 not discernible; tergum 9 nearly rectangular in lateral view; a pair of broad valvate gonocoxites 9 present, proximally with a pair of short and flat lobes, which are rounded at tip; ectoprocts paired and broad, nearly rectangular in lateral view; callus cerci not discernible.

*Etymology* The specific epithet “*lepidotricha*” refers to the presence of a row of scaly setae on the proximal section of forewing costal vein in the new species.

*Type material* Holotype: CAM BA-0018. Amber piece preserving a nearly complete female adult of *Xiaobabinskaia lepidotricha* gen. et sp. nov. It is polished in the form of a flattened elliptical cabochon, clear and transparent, with length × width 40.08 × 25.55 mm, height 9.37 mm.

### Phylogenetic analysis

**Figure 10:**
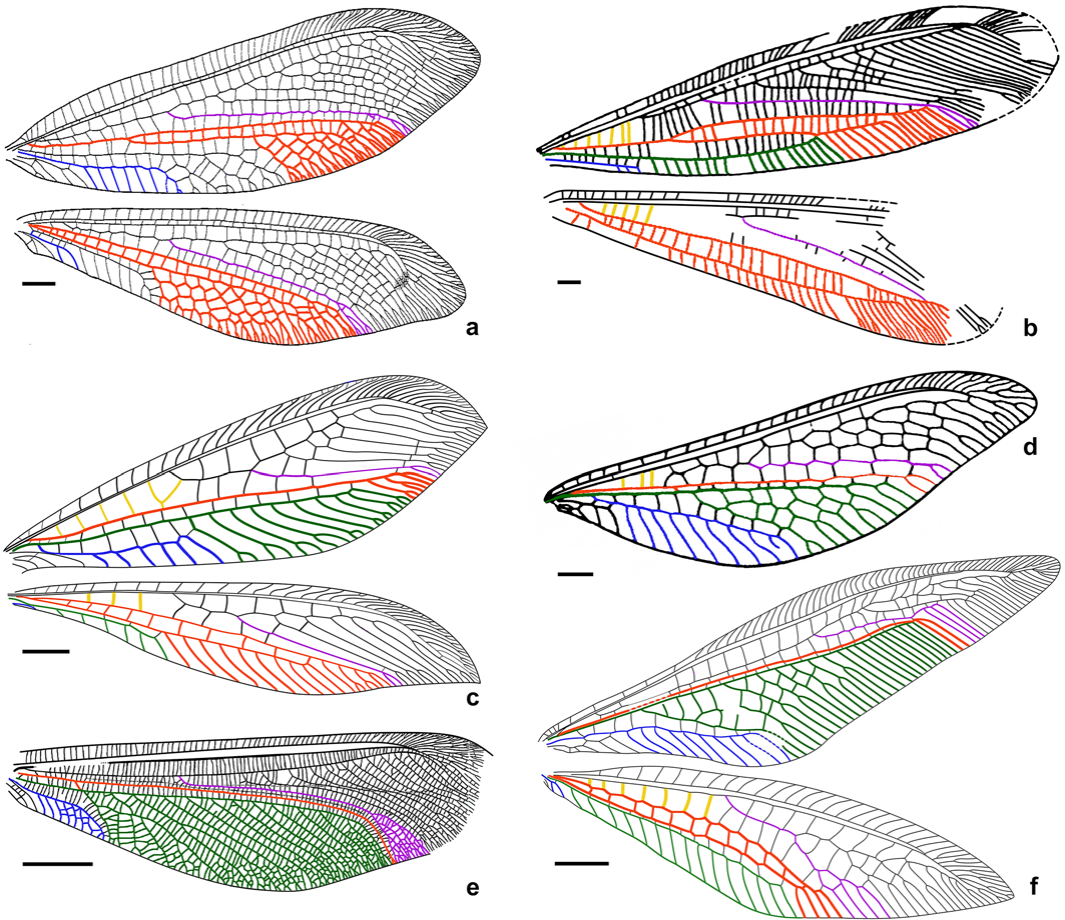
Comparison of wing venations among representatives of Myrmeleontoidea. (**a**) Nymphidae: *Nesydrion nigrinerve* Esben-Petersen^54^ (modified from New ^55^, republished with permission of Australian Journal of Zoology, from A revision of the Australian Nymphidae (Insecta: Neuroptera), New, T. R., 29, 707–750, 1981, permission conveyed through Copyright Clearance Center, Inc.). (**b**) Cratosmylidae: *Araripenymphes seldeni* Menon, Martins-Neto and Martill^18^ (drawn by XML); (**c**) Babinskaiidae: *Electrobabinskaia burmana* Lu et al.^6^ (drawn by XML); (**d**) Nemopteridae: *Pastranaia riojana* Orfila^56^ (forewing only, drawn by XML); (**e**) Palaeoleontidae: *Parapalaeoleon magus* Menon and Makarkin^47^ (forewing only, drawn by XML); (**f**) Myrmeleontidae: *Phylloleon stangei* Lu et al.^22^ (drawn by XML). Venations with presectoral crossveins (yellow), MA (purple), MP and oblique vein (red), CuA (green) and CuP (blue) highlighted. Scale bars = 1.0 mm (**b**, **c**); 2.0 mm (**a**, **d**, **f**); 5.0 mm (**e**).

**Figure 11:**
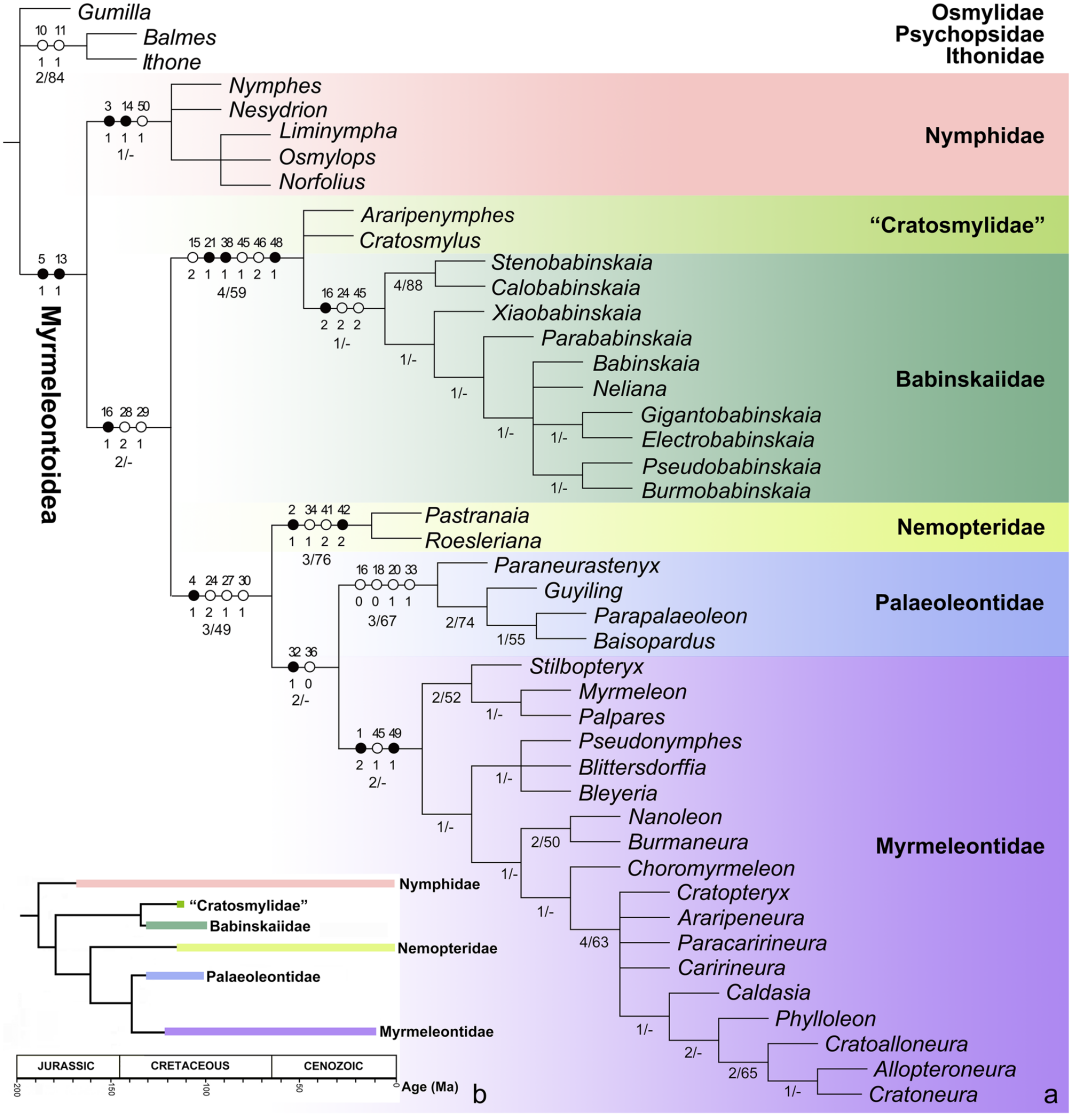
Phylogeny and evolutionary chronogram of extinct and extant Myrmeleontoidea. (**a**) Topology represents the strict consensus tree of the eight most parsimonious trees yielded from TNT v1.5 (www.zmuc.dk/public/phylogeny^52^ (see more detail information in supplementary material, Fig. S1). Unambiguous state changes of the morphological characters at the familial and higher level are shown on the tree. Black circle represents the homologous state and white circle represents the homoplasious state. Bremer support values/Bootstrap values are shown at relevant nodes. (**b**) Scheme of the interfamilial phylogeny of Myrmeleontoidea based on present result, thick lines indicate known geological distributions.

The maximum parsimony analysis in TNT yielded eight most parsimonious trees (MPTs) (length = 148, consistency index = 47, retention index = 81). The strict consensus tree and comparison of main venational characters with phylogenetic relevance are shown in Figs. 10, 11 and S1. The presently recovered monophyletic Myrmeleontoidea is supported by the absence of forewing nygmata (character 5:1) and the forewing ScP and RA terminating at or posteriad wing apex (character 13:1). Nymphidae is recovered to be the sister group of the clade including the remaining genera, and its autapomorphies comprise the prothorax slightly elongated anteriad procoxae (3:1), the presence of forewing thrydiate crossveins (character 14:1) and the bifid arolium (character 50:1). The monophyly of the latter clade is supported by the forewing RP + MA diverging slightly distal to wing base (16:1), and the forewing CuA branched near midpoint of wing with 10 or more branches (characters 28:2 and 29:1). Cratosmylidae and Babinskaiidae are clustered together, being sister to the lineage of Nemopteridae + (Myrmeleontidae + Palaeoleontidae). The monophyly of Cratosmylidae + Babinskaiidae is supported by the presence of more than two presectoral crossveins in both fore- and hind wings (characters 15:2 and 46:2), the reduction of crossveins on distal part of forewing radial space (21:1), the forewing CuP fused with A1 (38:1), hind wing RP + MA diverging from a position slightly distal to wing base (45:1), and the reduction of hind wing A2 and A3 (character 48:1). The two genera of Cratosmylidae did not form a monophylum. The assigned autapomorphies of Babinskaiidae include the RP + MA diverging from a position distinctly distal to wing base in both fore- and hind wings (15:2 and 45:2), and the single forewing MP (24:2). Within Babinskaiidae, *Stenobabinskaia* gen. nov. and *Calobabinskaia* gen. nov. are clustered together based on the presence of more than 10 presectoral crossveins in the forewing (character 15:3), the presence of trapezoidal forewing prehypostigmal cell (character 17:1), the forewing with distance between diverging point respectively of MA and RP1 twice as long as distance between diverging points respectively of RP1 and RP2 (22:1), the forewing MP1 terminating near wing apex (character 25:1), the forewing CuA branches marginally forked (character 31:1), and the extremely long forewing CuP (character 36:3). The remaining babinskaiid genera besides *Xiaobabinskaia* gen. nov. constitute another monophyletic group supported by the less crossveins in radial space (character 21:1) and the forewing CuP not fused with A1 (38:0), and together sister to *Xiaobabinskaia* gen. nov. by the zigzagged forewing CuP (37:1).

The monophyly of Nemopteridae + (Palaeoleontidae + Myrmeleontidae) is supported by the absence of trichosors (character 4:1), the single forewing MP (24:2), the presence of forewing oblique vein (character 27:1), and the subtriangular bra nching area of MP2 + CuA (character 30:1). The autapomorphies of Nemopteridae comprise the presence of prolonged rostrum (character 2:1), the closely spaced forewing CuA and CuP (character 34:1), and the strongly narrowed and elongated hind wing (characters 41:2 and 42:2). The monophylum including the genera of Myrmeleontidae and Palaeoleontidae is recovered based on the presence of short forewing CuA2 (character 32:1) and short hind wing CuP (character 36:0). The monophyly of Palaeoleontidae is supported based on the forewing RP + MA diverging from a positon near wing base (16:0), the short forewing hypostigmal cell (character 18:0), the forewing radial space with a Banksian line (character 20:1) and the pectinately branched forewing CuA2 with 3–4 branches (33:1). The monophyly of Myrmeleontidae is supported by the strongly dilated antennae (character 1:2), the origin of RP + MA slightly distal to wing base (character 45:1), and the presence of prolonged tibial spur (character 49:1). Within Myrmeleontidae, the extant three subfamilies form a monophyletic clade, being sister to another clade including Pseudonymphinae and Araripeneurinae. Araripeneurinae is nested in a clade including some Cretaceous antlion genera, i.e., *Choromyrmeleon* Ren and Guo^16^, *Nanoleon* Hu, Lu and Liu in Lu et al.^6^ and *Burmaneura* Huang et al.^17^.

The overall nodal supports are not strong if considering the Bremer support values/Bootstrap values, which is probably due to the large proportion of missing characters in the fossil taxa. However, weak support does not always mean unreliable relationships, as the monophyly of many groups, such as Nymphidae and Nemopteridae, were recovered based on a number of apomorphic character states although these groups received low nodal support.

## Discussion

### Monophyly of Babinskaiidae

Babinskaiidae is a well-defined fossil myrmeleontoid family by a series of adult characters (see familial diagnosis in “Systematic palaeontology”) although the larvae of this family have not yet been reported. However, the autapomorphy of this family is not conspicuous. Martins-Neto^13^ first proposed three apomorphic characters of this family: (1) the forewing MP2 + CuA1 (i.e., CuA in this paper) reaching the apical margin, (2) the origin of forewing RP (i.e., RP + MA in this paper) far from wing base, and (3) the zigzagged forewing CuP. Makarkin et al. (2017) refuted the above characters 1 and 3 to be apomorphic, and outlined four apomorphic characters, i.e., the origin of forewing RP far from wing base (the above character 2), the presence of presectoral crossveins, the single forewing MP, and the reduction of hind wing A2 and A3. Among these characters, only the last character is considered to be the autapomorphy of Babinskaiidae^7^. In Makarkin and Staniczek^10^, however, this autapomorphy was modified to be the very small anal space and the reduction of A3 in the hind wing because distinct hind wing A2 is found in *Parababinskaia makarkini* Hu et al.^8^ and *Electrobabinskaia burmana* Lu et al.^6,8^^.^

In the present result of phylogenetic analysis, some putative autapomorphies of Babinskaiidae mentioned above (e.g., the presence of more than two presectoral crossveins in both fore- and hind wings and the reduced hind wing A2 and A3) turned to be the autapomorphies of Cratosmylidae + Babinskaiidae. Cratosmylidae is composed of two genera from the Lower Cretaceous of Brazil, i.e., *Araripenymphes* Menon et al.^18^ and *Cratosmylus* Myskowiak et al.^7,19^. The former taxon is placed within Nymphidae^18,19^. This latter taxon was originally established as a subfamily of Osmylidae^19^, but later transferred to Nymphidae by Winterton et al.^20^. The two genera were considered to represent a separate family Cratosmylidae by Makarkin et al.^7^. Cratosmylidae appears to be a transitional lineage between Nymphidae and Babinskaiidae because it has an intermediate type of characters (Fig. 11). For example, Cratosmylidae and Nymphidae have a more or less proximal position of the forewing RP + MA origin and a deeply branched forewing MP, while Cratosmylidae and Babinskaiidae have several presectoral crossveins. Our result supports that *Araripenymphes* and *Cratosmylus* should not belong to Nymphidae (Fig. 11, S1). The closer relationship between Cratosmylidae and Babinskaiidae, as well as the paraphyly of the former family herein recovered, invokes another option that these two taxa may constitute a single family, with Cratosmylidae treated to be a subfamily of Babinskaiidae, as they share some similar apomorphic characters. Nevertheless, due to the scarcity of the cratosmylid fossils, we here retain the current classification until more materials are available for further evaluation.

### Phylogenetic position of Babinskaiidae

In the phylogenetic analysis of Myrmeleontoidea combining fossil and extant families^13^, Babinskaiidae was recovered to be the sister group of the clade including most myrmeleontoid families except Nymphidae, which is supported by the presence of forewing presectoral crossveins and forewing MP2 fused with CuA1. However, the forewing presectoral crossveins is absent in many genera of the families/subfamilies within the above clade, such as Araripeneurinae, Pseudonymphinae, Palaeoleontidae, etc. Besides, no oblique vein that is indicative of the fusion between forewing MP2 and CuA1 is found in any babinskaiids^6,7,21,22^. Our result is generally consistent with that in Martins-Neto^13^ concerning the position of Babinskaiidae + Cratosmylidae. However, the sister-group relationship between Babinskaiidae + Cratosmylidae and Nemopteridae + (Myrmeleontidae + Palaeoleontidae) is herein supported by the forewing RP + MA diverging from a position slightly distal to wing base, the forewing CuA initially branched near the midpoint of wing, and the forewing CuA with 10 or more branches. Nonetheless, this argument is also not strong enough because the second character state is also present in some genera of Nymphidae and the third character state is absent in most antlions. Given the complex and sometimes unpredictable evolutionary pattern of insect wing venations^23^, the presently used wing characters may not provide sufficient phylogenetic signal to resolve the higher phylogeny of Myrmeleontoidea, especially including many fossil taxa. However, the morphological arguments in Makarkin et al.^7^ placing Babinskaiidae together with Nymphidae in Nymphidoidae, i.e., the presence of trichosors and the completely separated forewing MP and CuA, are obviously attributed to the plesiomorphic condition in Neuroptera, which was also mentioned by these authors. Therefore, the phylogenetic position of Babinskaiidae herein recovered is still preferable, but awaits further evidence for corroboration.

Besides the adult characters, the larval morphology of Myrmeleontoidea also provides an important set of characters that are phylogenetically informative^24^. So far, no definite larva of Babinskaiidae has been reported. Badano et al.^25^ presented an interesting study focusing on the higher phylogeny of Myrmeleontiformia by using larval characters from both extant and fossil taxa. Notably, most fossil taxa sampled for the phylogenetic analysis in Badano et al.^25^ are from Kachin amber. Thus, this work is highly relevant to the present study and may provide additional evidence to infer the phylogenetic position of Babinskaiidae. Babinskaiidae is the most diverse and abundant group of Myrmeleontoidea from Kachin amber^1^. In the phylogeny recovered in Badano et al.^25^, the diverse Kachin amber myrmeleontoids without definite familial affiliations (e.g., *Electrocaptivus xui* Badano, Engel and Wang in Badano et al.^25^, *Burmitus tubulifer* Badano, Engel and Wang in Badano et al.^25^, *Adelpholeon lithophorus* Badano and Engel in Badano et al., 2018^25^, etc.) are assigned to be the stem-group Ascalaphidae + Myrmeleontidae, while there is no stem-group taxon or “side branch” found for Nymphidae as mentioned in Makarkin et al.^7,21^. Taphonomically, the diverse Kachin amber myrmeleontoid larvae that do not belong to any extant family may be associated with the extinct family Babinskaiidae, which is also diverse in species from the same deposit. Following this assumption, in the phylogenetic tree of Badano et al.^24^, Babinskaiidae is nested within the clade including Nemopteridae, Ascalaphidae, and Myrmeleontidae, which is partially concordant to the present result, although the identical position and monophyly of Babinskaiidae is not recovered as in our result. Of course, there are other possibilities on the identity of these larvae, such as those of the extinct Araripeneurinae that are also relatively basal to the extant antlions. Future in-depth study on the taxonomy of the Kachin amber myrmeleontoid larvae and their associations with the adult forms is important to resolve the deep phylogeny of Myrmeleontoidea.

### Diversification of Babinskaiidae

The new babinskaiids from Kachin amber highlight the early diversification of this Cretaceous lacewing lineage. Besides the rich species diversity, the Kachin amber babinskaiids, currently comprising nine genera and 10 species, display diverse morphological characters (Table S1). *Pseudobabinskaia martinsnetoi* (Lu et al.)^6^ is known to date as the smallest species of the family in body-size (forewing length ~ 9.0 mm), while *C. xiai* sp. nov. and *Gigantobabinskaia godunkoi* Makarkin and Staniczek^10^ are among the largest babinskaiids (forewing length over 22.0 mm)^6,10^. Specialized wing shape is also remarkable in some Kachin amber babinskaiids. For example, *C. xiai* sp. nov. and *S. punctata* sp. nov. possess distinctly narrow and elongate wings; *E. burmana*, *G. godunkoi*, and *X. lepidotricha* sp. nov. have modified hind wings that are distally falcate; and more peculiarly *Burmobabinskaia tenuis* Lu et al.^6^ has strongly narrowed hind wing similar to that of Nemopteridae^6,10^.

The morphological diversity of legs are highlighted by the differently modified tarsi and arolium. The tarsomeres are usually dilated in many babinskaiids, being indicative of an arboreal life mode, but the degree of development greatly varies among species^10^. In *G. godunkoi* all five metatarsomeres are strongly dilated with long hairs^10^. A similar trait is also present in *E. burmana*, but being slightly less developed^6^. In the other Burmese amber babinskaiids, such tarsal dilation is not as distinct as the former two species. However, in *C. xiai* sp. nov. the tarsomeres of all legs are densely setose and bear several long spinous, distally directed setae on the ventral surface. More interestingly, this species has bifid arolium in all legs, suggesting that this character state previously considered to be an autapomorphy of Nymphidae^26^ is convergently derived in other myrmeleontoid species. Additionally, in *C. xiai* sp. nov. there are some regularly spaced spinous setae on femora and tibiae, which seemingly function for predation or defense.

The scaly setae on the basal section of forewing costa in *X. lepidotrica* sp. nov. is another striking trait (Figs. 7, 8). This is not only the first report of scales in Babinskaiidae but also in all fossil and extant myrmeleontoids. Such scales especially resemble those in some beaded lacewings (Neuroptera: Berothidae). So far, function of the scales in Berothidae is completely unknown, however, in this family these scales are only found in females^27–29^. Thus, the scales in female beaded lacewings may be a kind of organs related to chemical communication for attracting or searching mates. Notably, the scales in babinskaiids herein reported are also present in female. Although the male of *X. lepidotrica* sp. nov. has not been found, if the scales are absent in males of this species, similar function of this trait involved in courtship as hypothesized in Berothidae may be inferred. Nevertheless, convincing evidence awaits discovery. Scales are also present on the wing eyespot of some derived subfamilies of Kalligrammatidae, which is thought to have certain optical function^30^. It is evident that they may be not similar in form and function to the presently reported scales in Babinskaiidae. In the nemopterid subfamily Crocinae, some extant species have a specialized structure called “bulla”, which is a group of silky hairs, sometimes ended as a small knob, on the fore- or hind wings^31^. This trait is confined to males and thought to be a kind of scent organ^31^. Similarly, the pilula axillaris or Eltringham organ is a club-like projection laterally with a tuft of setae from the hind wing margin, and it is also confined to male antlions. However, this structure is proposed to be a dispersing organ that could spread but not produce the sexual pheromones^32^. In some fossil species of the extinct chrysopoid family Mesochrysopidae there is a bunch of elongated hairs on the forewing^33^. Possibly, this trait also has a similar function as the scales or specialized hairs or organ in aforementioned myrmeleontoids, although whether it is present in only male or female or both sexes is unknown.

Finally, it is noteworthy to mention some previously unknown characters of the female genitalia in Babinskaiidae. In *C. xiai* sp. nov. the valvate female gonocoxites 9 bear some short dentoid processes along the ventral margin. In many antlions there are a number of stiff setae called digging setae on some female genital sclerites, such as gonocoxites 8, gonocoxites 9, and ectoprocts^34,35^. This is an adaptive trait for laying eggs on sand-like substrate^34,35^, which is probably associated with the fossorial life-style of some antlion larvae. In *X. lepidotrica* sp. nov. there is an additional lobe on anteroventral portion of the female gonocoxite 9, and this modification is similar to the hypocaudae in some berothid species^28^. In total, five types of female genitalia have thus far been described in Babinskaiidae (see Lu et al.^6^; Hu et al.^8^), suggesting disparate copulation or oviposition behaviors of these archaic myrmeleontoid species.

## Conclusion

The palaeofauna of Babinskaiidae from the mid-Cretaceous Kachin amber of Myanmar is represented by diverse genera and species with disparate morphological modifications. Our findings provide significant new morphological evidence for understanding the phylogenetic position and diversity of Babinskaiidae. In terms of the phylogenetic position of Babinskaiidae herein recovered, this Cretaceous family probably represents a transitional lineage between Nymphidae and the advanced myrmeleontoids. The Cretaceous radiation and recent decline of Babinskaiidae remains mysterious, awaiting discovery of palaeodiversity and phylogenetic study combining adult and larval characters.

## Material and methods

### Taxonomy

The amber specimens described herein are from the Hukawng Valley, Tanai Township, Myitkyina District, Kachin State, Myanmar (see Kania et al.^36^: Fig. 1). The age of this deposit is dated to be ~ 99 million years (the earliest Cenomanian) by U–Pb dating of zircons from the volcaniclastic matrix of the amber^37^.

The type specimens are deposited in the Nanjing Institute of Geology and Palaeontology (NIGP), Chinese Academy of Sciences, Nanjing; the Lingpoge Amber Museum (LAM), Shanghai; and the Century Amber Museum (CAM), Shenzhen.

Photographs and drawings were taken and made using a Leica M125C microscope system connecting with a Canon EOS 5D Mark IV camera system. The figures were prepared with Adobe Photoshop CS6. Terminology of wing venation generally follow Aspӧck et al.^38^ and Martins-Neto^39^. Terminology of genitalia follows Aspӧck and Aspӧck^40^.

Abbreviations used for wing veins are: A, anal vein; C, costa; Cu, cubitus; CuA, cubitus anterior; MP, media posterior; R, radius; RA, radius anterior; RP, radius posterior; ScA, subcosta anterior; ScP, subcosta posterior; ps, presectoral crossveins (i.e., r-mp crossveins).

All taxonomic acts established in this paper have been registered in ZooBank, together with the electronic publication: urn:lsid:zoobank.org:pub:605A4DCC-9ADE-473C-93BD-31878E03FA07.

### Phylogenetic analysis

Aiming to reveal the phylogenetic position of Babinskaiidae within Myrmeleontoidea, a phylogenetic analysis was performed based on samples from major fossil and extant lineages of Myrmeleontoidea. For the ingroup taxa in the present analysis, we sampled most known genera of Babinskaiidae except *Baisonelia* (only hind wings preserved) and *Pseudoneliana* (distal part of both wings not preserved). For Nymphidae, the stem-group of this family (*Liminympha* Ren and Engel^41^) as well as the extant representatives of Nymphinae (*Nesydrion* Gerstaecker^42^ and *Nymphes* Leach^43^) and Miodactylinae (*Osmylops* Banks^44^ and *Norfolius* Navás^45^) were selected. Two nymphid-like genera from the Lower Cretaceous of Brazil, i.e., *Araripenymphes* and *Cratosmylus*, which were considered to represent a separate family Cratosmylidae by Makarkin et al.^7^, were selected. Three genera of the extinct family Palaeoleontidae, i.e., *Baisopardus* Ponomarenko^4^, *Paraneurastenyx* Martins-Neto^46^, and *Parapalaeoleon* Menon and Makarkin^47^, were selected because the species of these genera have better preserved morphological characters than the other species of this poorly known myrmeleontoid fossil family. The other sampled ingroup taxa are the same to those in Lu et al.^22^. Notably, the genera *Stilbopteryx* and *Palpares* are assigned to the subfamily Ascalaphinae in Machado et al.^48^, representing ascalaphids in the present sampling. We selected *Gumilla* Navás^49^ (Osmylidae), *Ithone* Newman^50^ (Ithonidae) and *Balmes* Navás^51^ (Psychopsidae) as the outgroup taxa.

Morphological characters used in the phylogenetic analysis are mainly from Martins-Neto^13,14^ and Lu et al.^22^ with some modification, comprising a total of 54 adult characters (see Note S1). Unknown characters were coded as “?”, while inapplicable characters were coded as “-”. The data matrix is also given in Table S2. All characters were treated as unordered and with equal weight. We analyzed the dataset using TNT v1.5^52^ with an initial Traditional search (Starting trees: 100 repls, TBR, trees to save per replication: 10). Bremer support values and Bootstrap values were calculated with the function implemented in TNT (Setting for Bremer support values calculation: TBR from existing trees, retain trees suboptimal by 10 steps; setting for Bootstrap values calculation: traditional search, number of replicates: 1000). Character states were mapped on the strict consensus tree (MPT) using WinClada ver. 1.00.08^53^, showing only unambiguous changes.

## Supplementary Information

Supplementary Information 1.

Supplementary Information 2.
