# Supplementary material for: New Cretaceous antlion-like lacewings promote a phylogenetic reappraisal of the extinct myrmeleontoid family Babinskaiidae
Source: Sci Rep. 2021 Aug 12;11:16431. doi: 10.1038/s41598-021-95946-z (PMC8361207; doi:10.1038/s41598-021-95946-z)
Supplement: Supplementary file 1 — Supplementary Information 1. [file 41598_2021_95946_MOESM1_ESM.doc]

#NEXUS

[written Mon Jun 21 14:30:34 CST 2021 by Mesquite version 3.51 (build 898) at PC/172.17.59.88]

BEGIN TAXA;

TITLE Taxa;

DIMENSIONS NTAX=44;

TAXLABELS

Gumilla Ithone Balmes Nesydrion Nymphes Norfolius Osmylops Liminympha Cratosmylus Ararinymphes Neliana Parababinskaia Babinskaia Burmobabinskaia Electrobabinskaia Pseudobabinskaia Gigantobabinskaia Calobabinskaia Stenobabinskaia Xiaobabinskaia Roesleriana Pastranaia Choromyrmeleon Caririneura Cratoneura Cratopteryx Paracaririneura Araripeneura Caldasia Cratoalloneura Allopteroneura Phylloleon Burmaneura Nanoleon Bleyeria Blittersdorffia Pseudonymphes Palpares Stilbopteryx Myrmeleon Guyiling Baisopardus Parapalaeoleon Paraneurastenyx

;

END;

BEGIN CHARACTERS;

TITLE Character_Matrix;

DIMENSIONS NCHAR=54;

FORMAT DATATYPE = STANDARD GAP = - MISSING = ? SYMBOLS = " 0 1 2 3 4";

CHARSTATELABELS

1 antennae / filiform slightly_dilated strongly_dilated,

2 Head_with_prolonged_rostrum / absent present,

3 Head_with_prothorax / short slightly_elongate,

4 trichosors / present absent,

5 FW_nygmata / present absent,

6 FW_shape / distally_not_strongly_narrowed distally_strongly_narrowed,

7 'FW length: width' / 2.0_times_as_long_as_wide slightly_longer_than_broad at_least_3.0_times_as_long_as_wide,

8 Crossveins / normal dense,

9 FW_costal_space / not_strongly_narrowed_basally strongly_narrowed_basally,

10 FW_Costal_crossveins / basally_simple basally_forked,

11 FW_humeral_veinet / simple recurrent_and_branched,

12 FW_Sc_and_RA / not_fused_distally fused_distally triplica,

13 FW_ScP_and_RA_terminating / anteriad_wing_apex at_or_posteriad_wing_apex,

14 Thyridiate / absent present,

15 FW_ps / absent 'present, 1' 'present, more than 2' 'present, at least 10',

16 'FW RP+MA' / wing_base slightly_distad distinctly_distad,

17 FW_prehypostigmal_cell / rectangular trapezoidal,

18 FW_hc / short long,

19 FW_intraradial_cell / short long,

20 FW_radial_space / without_Banksian_line with_a_Banksian_line,

21 FW_crossveins_in_radial_space / developed_distally slightly_reduced reduced,

22 'FW MA to RP1: RP1 to RP2' / 1 2,

23 FW_MA / dichotomously_branched_or_simple pectinately_branched,

24 FW_MP / deeply_forked shallowly_forked single,

25 FW_MP1_terminating / middle_of_apex_margin near_wing_apex,

26 FW_MP1_branches / not_pectinate pectinate,

27 Oblique_vein / absent present,

28 FW_CuA_branches / '1-4' '5-9' '10-',

29 FW_CuA_branched / 'at distal 1/3' near_midpoint,

30 FW_CuA_branching_area / not_triangular subtriangular,

31 FW_CuA / not_forked forked,

32 FW_CuA2 / long short,

33 FW_CuA2 / bifurcated 'pectinated, 3-4' 'pectinated, 6',

34 FW_MP_and_CuA / not_close close,

35 FW_CuA2_origin / 'distad origin of RP+MA' 'proximad origin of RP+MA',

36 FW_CuP / short 'long, not proximal 1/3' 'long, near midpoint' 'extremely long, posteriad midpoint',

37 FW_CuP / straight zigzagged,

38 FW_CuP / not_fused_with_A1_distally fused_with_A1_distally,

39 FW_A1 / shallowly_forked deeply_forked pectinate simple,

40 HW / not_strongly_tapering_distad tapering_distad,

41 HW / not_narrowed slightly_narrowed strongly_narrowed,

42 HW / not_strongly_elongated slightly_elongated strongly_elongated,

43 HW_costal_space / narrower_than_FW_costal_space wider_than_FW_costal_space,

44 HW_costal_margin / straight arched,

45 'HW RP+MA origin' / basad slightly_distad distinctly_distad,

46 HW_ps / absent 'present, 1' 'present, more than 2',

47 'HW 1r-m' / sigmoid straight_or_reduced,

48 HW_A2_and_A3 / preserved reduced,

49 tibial_spur / nor_prolonged prolonged,

50 Foreleg_arolium / single bibled,

51 Male_gx9 / basally_external basally_internal,

52 Male_gx9_and_gx11 / separate forming_a_complex,

53 Female_trichobothria / rosette absent,

54 Female_S6 / not_elongated apparently_elongated ;

MATRIX

Gumilla 0000002000010000010000000-0000100001102000000010000000

Ithone 0000000001100000010000000-01000--001001000000000001000

Balmes 0000001001120000010020000-01000--001001000000010001000

Nesydrion 0010102000011100010000000-00000--001000000000210011000

Nymphes 00101020000111000100010(0 1)0-01110--001000000000010011000

Norfolius 0010102000011100010000000-00000--002002000000010011000

Osmylops 0010102000011100010000000-00000--002002000000010011000

Liminympha 0?10102000111?00010000000-01000--00210200000001???????

Cratosmylus ??00000000011021000010?00-0?10???001?1-000001211??????

Ararinymphes ??001020000110210?0010000-02100--0100??00000121???????

Neliana 00001020000110220100200(1 2)010(1 2)100--0111000000022110?????

Parababinskaia 000010200001102201002002110(1 2)100--001100000002211001?00

Babinskaia 0000102000011022010020020101100--0111000000022110?????

Burmobabinskaia 00001??0000??0220?002002110?100--011103?2?002211001???

Electrobabinskaia 0000102000011022010020020102100--011103100002211001?01

Pseudobabinskaia 0000102000011022010020021101100--01110300000221100??01

Gigantobabinskaia 0?001??0010??022?????0?2?10?1?0--01110310000221100????

Calobabinskaia 00001020000110321100210211011010000301-00000221?01????

Stenobabinskaia 00001020000110321100110211021010000301-0000022110?1???

Xiaobabinskaia 0000102000011022010010020102100--00111-10000221100???0

Roesleriana 1101102000011001010000020012110--1010??-2200001-001000

Pastranaia 010110200001102101000002001111100101000-2200001-001000

Choromyrmeleon 20011020000110010110000200111111000100300000101???????

Caririneura 20011020000110011110001200011110011100211000221???????

Cratoneura 2001112010011001111101120002110--1110??10011101???????

Cratopteryx 2001102000011001111000120011111001010??10000101???????

Paracaririneura 200110200001100111000112000111100101003???????????????

Araripeneura 20011020000110011110011200011110011100310000101???????

Caldasia 200110200001100111100012000??????11????10011??????????

Cratoalloneura 20011120100110011111011200111110011100010011101???????

Allopteroneura 200111201001?0011111011200121110011100010011101?10????

Phylloleon 200111201001100111000012001(1 2)111001010001(0 1)(0 1)11221010??10

Burmaneura 20011?20000??001????0??20?01110--0000030000011101?????

Nanoleon 20011020000110110100000200011111000000300000111010??10

Bleyeria 20011020000110010000000200111111100000000000101???????

Blittersdorffia 200110200001100101000102001(1 2)1111100000000000101???????

Pseudonymphes 20001020000110010100000(0 2)001(0 1)11111000002000001010??????

Palpares 200110200001102101000002001211110110000000001210101110

Stilbopteryx 200110200001102101000002001211110100000000001110101110

Myrmeleon 200110200001102101000002001211110110000000001210101110

Guyiling 100110?10001?0000?0101?20012111110000????000001?0?????

Baisopardus 0001102100011000000101020012111120000030000000100?????

Parapalaeoleon 00011021000110000001010200121111200000300?????????????

Paraneurastenyx ??01102000011000000100?2001211111000000???????????????

;

END;
