# Supplementary material for: New Cretaceous antlion-like lacewings promote a phylogenetic reappraisal of the extinct myrmeleontoid family Babinskaiidae
Source: Sci Rep. 2021 Aug 12;11:16431. doi: 10.1038/s41598-021-95946-z (PMC8361207; doi:10.1038/s41598-021-95946-z)
Supplement: Supplementary file 2 — Supplementary Information 2. [file 41598_2021_95946_MOESM2_ESM.doc]

Supplementary material for:

**New Cretaceous antlion-like lacewings promote a phylogenetic reappraisal of the extinct myrmeleontoid family Babinskaiidae**

Xiumei Lu^1*^, Bo Wang^2^, Xingyue Liu^3*^

**Contents**

**Table S1. Checklist of Babinskaiidae.**

**Figure S1. Strict consensus tree of the eight most parsimonious trees yielded from TNT v1.5 (www.zmuc.dk/public/phylogeny)^52^.** Unambiguous morphological character states are shown on the tree with a black circle as the homologous state and a white circle as the homoplasious state. Bremer support values/Bootstrap values are shown at relevant nodes.

**Note S1. Morphological character matrix used in the phylogenetic analysis.**

**Table S2. List of characters coded for the phylogenetic analysis.**

**File S1. Data matrix in the phylogenetic analysis.**

**Supplementary references** (number continued with that in References section in the main text).

**Table S**1. Checklist of Babinskaiidae.

| **No.** | **Species** | **Age** | **Locality** |
| --- | --- | --- | --- |
| 1 | *Baisonelia vitimica* Ponomarenko, 1992 | Lower Cretaceous, Barremian | Zaza Formation, Russia |
| 2 | *Babinskaia formosa* Martins-Neto & Vulcano, 1989 | Lower Cretaceous, Upper Aptian | Crato Formation, Brazil |
| 3 | *Babinskaia pulchra* Martins-Neto & Vulcano, 1989 | Lower Cretaceous, Upper Aptian | Crato Formation, Brazil |
| 4 | *Neliana maculata* (Martins-Neto & Vulcano, 1989) | Lower Cretaceous, Upper Aptian | Crato Formation, Brazil |
| 5 | *Neliana impolluta* Martins-Neto, 1997 | Lower Cretaceous, Upper Aptian | Crato Formation, Brazil |
| 6 | *Parababinskaia elegans* Makarkin, Heads & Wedmann, 2017 | Lower Cretaceous, Upper Aptian | Crato Formation, Brazil |
| 7 | *Burmobabinskaia tenuis* Lu et al., 2017 | mid-Cretaceous, lowest Cenomanian | Tanai, Myitkyina, Kachin, Myanmar |
| 8 | *Calobabinskaia xiai* gen. et sp. nov. | mid-Cretaceous, Lowest Cenomanian | Tanai, Myitkyina, Kachin, Myanmar |
| 9 | *Electrobabinskaia burmana* Lu et al., 2017 | mid-Cretaceous, Lowest Cenomanian | Tanai, Myitkyina, Kachin, Myanmar |
| 10 | *Gigantobabinskaia godunkoi* Makarkin & Staniczek, 2019 | mid-Cretaceous, Lowest Cenomanian | Tanai, Myitkyina, Kachin, Myanmar |
| 11 | *Stenobabinskaia punctata* gen. et sp. nov. | mid-Cretaceous, Lowest Cenomanian | Tanai, Myitkyina, Kachin, Myanmar |
| 12 | *Parababinskaia makarkini* Hu et al., 2018 | mid-Cretaceous, Lowest Cenomanian | Tanai, Myitkyina, Kachin, Myanmar |
| 13 | *Parababinskaia douteaui* Ngô-Muller et al., 2020 | mid-Cretaceous, Lowest Cenomanian | Tanai, Myitkyina, Kachin, Myanmar |
| 14 | *Pseudobabinskaia martinsnetoi* (Lu et al., 2017) | mid-Cretaceous, Lowest Cenomanian | Tanai, Myitkyina, Kachin, Myanmar |
| 15 | *Pseudoneliana* *makarkini* Huang, André & Dany, 2019 | mid-Cretaceous, Lowest Cenomanian | Tanai, Myitkyina, Kachin, Myanmar |
| 16 | *Xiaobabinskaia lepidotricha* gen. et sp. nov. | mid-Cretaceous, Lowest Cenomanian | Tanai, Myitkyina, Kachin, Myanmar |

**Figure S1. Strict consensus tree of the eight most parsimonious trees yielded from TNT v1.5 (www.zmuc.dk/public/phylogeny)^52^.** Unambiguous morphological character states are shown on the tree with a black circle as the homologous state and a white circle as the homoplasious state. Bremer support values/Bootstrap values are shown at relevant nodes.


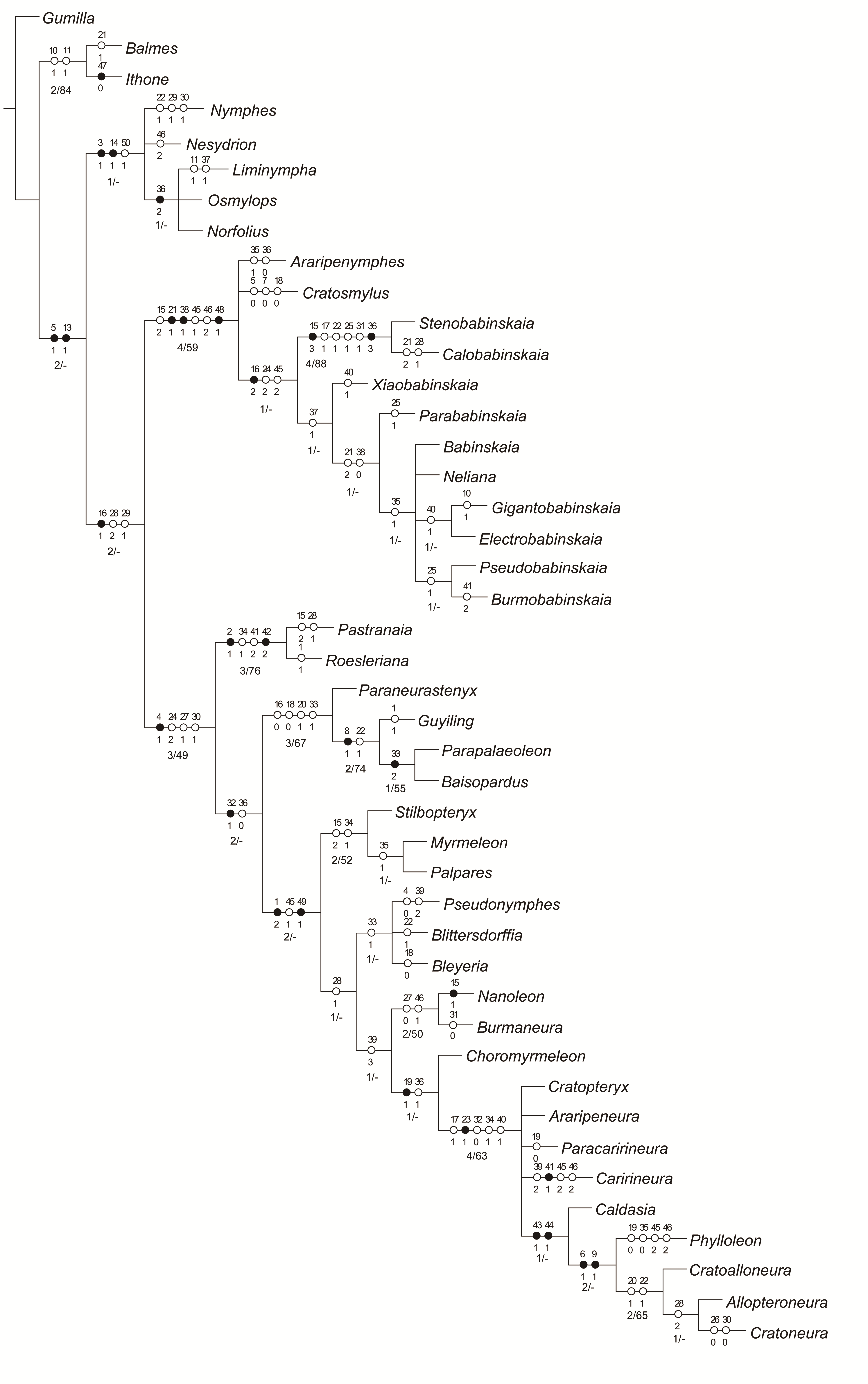


**Note S1. List of characters coded for the phylogenetic analysis.**

1. Antenna: (0) filiform, not dilated distad (Fig. 10g5^12^); (1) filiform, but slightly dilated distad (Fig. 10g1^12^); (2) distinctly clubbed (Fig. 10g2^12^).
2. Head with prolonged rostrum: (0) absent (Fig. 2b^6^); (1) present (Figs. 1898–1899^31^).
3. Prothorax: (0) not elongated anterior to procoxae (Figs. 13–20^57^); (1) elongated anterior to procoxae (Fig. 3^26^).
4. Trichosors: (0) present (Figs. 2, 5, 7); (1) largely reduced or absent (Fig. 10f).
5. Nygmata: (0) present (Fig. 6^19^); (1) absent (Fig. 10).
6. Forewing shape: (0) distally not strongly narrowed (Fig. 10a–e); (1) distally strongly narrowed (Fig. 10f).
7. Forewing: (0) 2.0 times as long as wide (Fig. 48^58^); (1) slightly longer than wide (Figs. 13–20^57^); (2) at least 3.0 times as long as wide (Fig. 10).
8. Crossveins: (0) normal (Fig. 10a–e); (2) dense (Fig. 10e).
9. Forewing costal space: (0) not strongly narrowed proximally (Fig. 10a–e); (1) strongly narrowed proximally (Fig. 10f).
10. Forewing costal crossveins on proximal part of costal space: (0) simple (Fig. 10); (2) forked (Fig. 48^58^).
11. Forewing humeral veinlet: (0) simple (Fig. 10); (0) recurrent and branched (Fig. 48^58^).
12. Forewing Sc and RA: (0) not fused distally (Fig. 48^58^); (1) fused distally (Fig. 10); (2) allied together with RP into a triplica (Figs. 13–20^57^).
13. Forewing ScP and RA terminating: (0) anteriad wing apex (Fig. 48^58^); (1) at or posteriad wing apex (Fig. 10).
14. Forewing thyridiate crossveins: (0) absent (Fig. 10b–f); (1) present (Fig. 10a).
15. Forewing presectoral crossveins: (0) absent (Fig. 10a, e–f); (1) present, only one (Fig. 10^22^); (2) present, more than 2 (Fig. 10b–d); (3) present, more than 10 (Figs. 2, 5).
16. Forewing RP+MA: (0) diverging almost from wing base (Fig. 10a, e); (1) diverging slightly distad wing base (Fig. 10b, d, f); (2) diverging from a position apparently distad wing base (Figs. 2, 5, 7, 10c).
17. Forewing prehypostigmal cell: (0) rectangular (Fig. 10a–e); (1) trapezoidal (Fig. 10f).
18. Forewing hypostigmal cell: (0) short (Fig. 10a–b, e); (1) long (Fig. 10c–d, f).
19. Forewing infra radial cell: (0) short (Fig. 10); (1) long (Fig. 2^22^).
20. Forewing branching area of RP+MA: (0) without longitudinally directed outer gradate series of crossveins (Fig. 10a–d, f); (1) with longitudinally directed outer gradate series of crossveins (Fig. 10e; Fig. 2^22^).
21. Forewing crossvein of branching area of RP+MA: (0) present throughout this region (Fig. 10a, d, e–f); (1) slightly reduced (Fig. 10b); (2) absent on distal half of this region (Fig. 10c).
22. Forewing with distance between diverging points respectively of MA and RP1: (0) as long as distance between diverging points respectively of RP1 and RP2 (Fig. 10); (1) at least twice as long as distance between diverging points respectively of RP1 and RP2 (Figs. 2, 5; Fig. 2^22^).
23. Forewing MA: (0) dichotomously branched or simple (Fig. 10a–e); (1) pectinately branched (Fig. 10f).
24. Forewing MP: (0) deeply forked (Fig. 10a–b); (1) shallowly forked (Fig. 8^7^); (2) single (Fig. 10c–f).
25. Forewing MP1 terminating: (0) far from wing apex (Fig. 7); (1) near wing apex (Figs. 2, 5).
26. Forewing single MP branches: (0) dichotomously branched or simple (Fig. 10d–f); (1) pectinate (Fig. 10c).
27. Forewing with base of MP2 (oblique vein): (0) absent (Fig. 10c); (1) present (Fig. 10d–e; Fig. 2^22^).
28. Forewing, number of CuA branches: (0) 1–4 (Fig. 10a); (1) 5–9 (Fig. 10d); (2) at least 10 (Fig. 10b–c, e–f). Considering the boundary between CuA and CuP is controversial, the boundary of CuA and CuP in Babinskaiidae follows that in Hu et al (see Fig. 3A^8^).
29. Forewing CuA branched: (0) at distal 1/3 (Fig. 10a); (1) near or proximad midpoint (Fig. 10b–f).
30. Forewing MP2+CuA branching area: (0) not triangular (Fig. 10a–c); (1) subtriangular (Fig. 10d–f).
31. Forewing CuA: (0) not forked (Fig. 10a–c); (1) forked (Figs. 2, 5, 10d–f).
32. Foweing CuA2: (0) longer than CuA1 branches (Fig. 10d, f); (1) shorter than CuA1 branches (Fig. 10e).
33. Forewing CuA2: (0) bifurcated (Fig. 10d, f); (1) with 3-4 pectinate branches (Fig. 13^21^); (2) with 6 pectinate branches (Fig. 10e).
34. Forewing MP1 and MP2+CuA: (0) not closely spaced (Fig. 10c, e); (1) closely spaced (Fig. 10d, f).
35. Forewing CuA2 origin: (0) distad origin of RP+MA (Fig. 10d–f); (1) proximad origin of RP+MA (Fig. 2^22^).
36. Forewing CuP: (0) short (Fig. 10b, e); (1) long, terminating near proximal 1/3 of hind margin (Fig. 10a, c); (2) long, terminating anteriad midpoint of hind margin (Fig. 10d, f); (3) extremely long, terminating posteriad midpoint of hind margin (Figs. 2, 5).
37. Forewing CuP: (0) straight (Fig. 10a–b, d–f); (1) zigzagged (Figs. 8, 10c).
38. Forewing CuP: (0) not fused with A1 distally (Fig. 10); (1) fused with A1 distally (Figs. 2, 5, 7).
39. Forewing A1: (0) shallowly bifurcated (Fig. 10a, d, f); (1) deeply bifurcated (Fig. 48^58^); (2) pectinate (Fig. 109^55^); (3) simple (Fig. 10c).
40. Hind wing: (0) not strongly tapering distad (Fig. 10a, b); (1) strongly tapering distad (Fig. 10c, f).
41. Hind wing: (0) not narrowed (Fig. 10a, b); (1) slightly narrowed (Fig. 7^22^); (2) strongly narrowed (Fig. 1^56^).
42. Hind wing: (0) not elongated, shorter than forewing (Fig. 10a–c, f); (1) slightly elongated, slightly longer than forewing (Fig. 7^22^); (2) strongly elongated, apparently longer than forewing ( Fig. 1^56^).
43. Hind wing costal space: (0) narrower than forewing costal space (Fig. 10a–c); (1) wider than forewing costal space (Fig. 10f; Fig. 2^22^).
44. Hind wing costal margin: (0) straight (Fig. 10a–c); (1) arched (Fig. 10f).
45. Hind wing RP+MA: (0) diverging almost from wing base (Fig. 10a); (1) diverging from a position slightly distad wing base (Fig. 2^22^); (2) diverging from a position near midpoint of wing (Fig. 10c, f).
46. Hind wing presectorial crossveins: (0) absent (Fig. 10a); (1) present, only one (Fig. 10^22^); (2) present, more than 2 (Fig. 10b–c, f).
47. Hind wing with stem of MA: (0) sigmoid (Fig. 48^58^); (1) straight or reduced (Fig. 10a–c, f).
48. Hind wing A2 and A3: (0) preserved (Fig. 10a); (1) reduced (Fig. 10c).
49. Tibial spur: (0) small or reduced (Fig. 1g); (1) long and strout (Fig. 11^22^).
50. Foreleg arolium: (0) simple (Fig. 1d^8^); (1) bilobed (Fig. 1f).
51. Male gonocoxites 9: (0) present as a pair of large external sclerites (Fig. 3d^59^); (1) present as a pair of relatively small and internal sclerites (Fig. 2g^6^).
52. Male gonocoxites 9 and gonocoxites 11: (0) not associated tightly into a complex structure (Figs. 88–89^40^); (1) associated tightly into a complex structure (Figs. 94–96^40^).
53. Female trichobothria: (0) rosette (Fig. 4^6^); (1) absent (Fig. 3^22^).
54. Female sternum 6: (0) posteriorly without elongated processes (Fig. 7c^8^); (1) posteriorly with elongated processes (Fig. 7a–b^8^).

**Table S**2. Morphological character matrix used in the phylogenetic analysis.

|  |  | 01 | 02 | 03 | 04 | 05 | 06 | 07 | 08 | 09 | 10 | 11 | 12 | 13 | 14 | 15 | 16 | 17 | 18 | 19 | 20 | 21 | 22 | 23 | 24 | 25 | 26 | 27 |
| --- | --- | --- | --- | --- | --- | --- | --- | --- | --- | --- | --- | --- | --- | --- | --- | --- | --- | --- | --- | --- | --- | --- | --- | --- | --- | --- | --- | --- |
| 1 | *Gumilla* | 0 | 0 | 0 | 0 | 0 | 0 | 2 | 0 | 0 | 0 | 0 | 1 | 0 | 0 | 0 | 0 | 0 | 1 | 0 | 0 | 0 | 0 | 0 | 0 | 0 | - | 0 |
| 2 | *Ithone* | 0 | 0 | 0 | 0 | 0 | 0 | 0 | 0 | 0 | 1 | 1 | 0 | 0 | 0 | 0 | 0 | 0 | 1 | 0 | 0 | 0 | 0 | 0 | 0 | 0 | - | 0 |
| 3 | *Balmes* | 0 | 0 | 0 | 0 | 0 | 0 | 1 | 0 | 0 | 1 | 1 | 2 | 0 | 0 | 0 | 0 | 0 | 1 | 0 | 0 | 1 | 0 | 0 | 0 | 0 | - | 0 |
| 4 | *Nesydrion* | 0 | 0 | 1 | 0 | 1 | 0 | 2 | 0 | 0 | 0 | 0 | 1 | 1 | 1 | 0 | 0 | 0 | 1 | 0 | 0 | 0 | 0 | 0 | 0 | 0 | - | 0 |
| 5 | *Nymphes* | 0 | 0 | 1 | 0 | 1 | 0 | 2 | 0 | 0 | 0 | 0 | 1 | 1 | 1 | 0 | 0 | 0 | 1 | 0 | 0 | 0 | 1 | 0 | 0&1 | 0 | - | 0 |
| 6 | *Norfolius* | 0 | 0 | 1 | 0 | 1 | 0 | 2 | 0 | 0 | 0 | 0 | 1 | 1 | 1 | 0 | 0 | 0 | 1 | 0 | 0 | 0 | 0 | 0 | 0 | 0 | - | 0 |
| 7 | *Osmylops* | 0 | 0 | 1 | 0 | 1 | 0 | 2 | 0 | 0 | 0 | 0 | 1 | 1 | 1 | 0 | 0 | 0 | 1 | 0 | 0 | 0 | 0 | 0 | 0 | 0 | - | 0 |
| 8 | *Liminympha* | 0 | ? | 1 | 0 | 1 | 0 | 2 | 0 | 0 | 0 | 1 | 1 | 1 | ? | 0 | 0 | 0 | 1 | 0 | 0 | 0 | 0 | 0 | 0 | 0 | - | 0 |
| 9 | *Cratosmylus* | ? | ? | 0 | 0 | 0 | 0 | 0 | 0 | 0 | 0 | 0 | 1 | 1 | 0 | 2 | 1 | 0 | 0 | 0 | 0 | 1 | 0 | ? | 0 | 0 | - | 0 |
| 10 | *Araripenymphes* | ? | ? | 0 | 0 | 1 | 0 | 2 | 0 | 0 | 0 | 0 | 1 | 1 | 0 | 2 | 1 | 0 | ? | 0 | 0 | 1 | 0 | 0 | 0 | 0 | - | 0 |
| 11 | *Neliana* | 0 | 0 | 0 | 0 | 1 | 0 | 2 | 0 | 0 | 0 | 0 | 1 | 1 | 0 | 2 | 2 | 0 | 1 | 0 | 0 | 2 | 0 | 0 | 1&2 | 0 | 1 | 0 |
| 12 | *Parababinskaia* | 0 | 0 | 0 | 0 | 1 | 0 | 2 | 0 | 0 | 0 | 0 | 1 | 1 | 0 | 2 | 2 | 0 | 1 | 0 | 0 | 2 | 0 | 0 | 2 | 1 | 1 | 0 |
| 13 | *Babinskaia* | 0 | 0 | 0 | 0 | 1 | 0 | 2 | 0 | 0 | 0 | 0 | 1 | 1 | 0 | 2 | 2 | 0 | 1 | 0 | 0 | 2 | 0 | 0 | 2 | 0 | 1 | 0 |
| 14 | *Burmobabinskaia* | 0 | 0 | 0 | 0 | 1 | ? | ? | 0 | 0 | 0 | 0 | ? | ? | 0 | 2 | 2 | 0 | ? | 0 | 0 | 2 | 0 | 0 | 2 | 1 | 1 | 0 |
| 15 | *Electrobabinskaia* | 0 | 0 | 0 | 0 | 1 | 0 | 2 | 0 | 0 | 0 | 0 | 1 | 1 | 0 | 2 | 2 | 0 | 1 | 0 | 0 | 2 | 0 | 0 | 2 | 0 | 1 | 0 |
| 16 | *Pseudobabinskaia* | 0 | 0 | 0 | 0 | 1 | 0 | 2 | 0 | 0 | 0 | 0 | 1 | 1 | 0 | 2 | 2 | 0 | 1 | 0 | 0 | 2 | 0 | 0 | 2 | 1 | 1 | 0 |
| 17 | *Gigantobabinskaia* | 0 | ? | 0 | 0 | 1 | ? | ? | 0 | 0 | 0 | 0 | ? | ? | 0 | 2 | 2 | ? | ? | ? | ? | ? | 0 | ? | 2 | ? | 1 | 0 |
| 18 | *Calobabinskaia* | 0 | 0 | 0 | 0 | 1 | 0 | 2 | 0 | 0 | 0 | 0 | 1 | 1 | 0 | 3 | 2 | 1 | 1 | 0 | 0 | 2 | 2 | 0 | 2 | 1 | 1 | 0 |
| 19 | *Stenobabinskaia* | 0 | 0 | 0 | 0 | 1 | 0 | 2 | 0 | 0 | 0 | 0 | 1 | 1 | 0 | 3 | 2 | 1 | 1 | 0 | 0 | 1 | 2 | 0 | 2 | 1 | 1 | 0 |
| 20 | *Xiaobabinskaia* | 0 | 0 | 0 | 0 | 1 | 0 | 2 | 0 | 0 | 0 | 0 | 1 | 1 | 0 | 2 | 2 | 0 | 1 | 0 | 0 | 1 | 0 | 0 | 2 | 0 | 1 | 0 |
| 21 | *Roesleriana* | 1 | 1 | 0 | 1 | 1 | 0 | 2 | 0 | 0 | 0 | 0 | 1 | 1 | 0 | 0 | 1 | 0 | 1 | 0 | 0 | 0 | 0 | 0 | 2 | 0 | 0 | 1 |
| 22 | *Pastranaia* | 0 | 1 | 0 | 1 | 1 | 0 | 2 | 0 | 0 | 0 | 0 | 1 | 1 | 0 | 2 | 1 | 0 | 1 | 0 | 0 | 0 | 0 | 0 | 2 | 0 | 0 | 1 |
| 23 | *Choromyrmeleon* | 2 | 0 | 0 | 1 | 1 | 0 | 2 | 0 | 0 | 0 | 0 | 1 | 1 | 0 | 0 | 1 | 0 | 1 | 1 | 0 | 0 | 0 | 0 | 2 | 0 | 0 | 1 |
| 24 | *Caririneura* | 2 | 0 | 0 | 1 | 1 | 0 | 2 | 0 | 0 | 0 | 0 | 1 | 1 | 0 | 0 | 1 | 1 | 1 | 1 | 0 | 0 | 0 | 1 | 2 | 0 | 0 | 0 |
| 25 | *Cratoneura* | 2 | 0 | 0 | 1 | 1 | 1 | 2 | 0 | 1 | 0 | 0 | 1 | 1 | 0 | 0 | 1 | 1 | 1 | 1 | 1 | 0 | 1 | 1 | 2 | 0 | 0 | 0 |
| 26 | *Cratopteryx* | 2 | 0 | 0 | 1 | 1 | 0 | 2 | 0 | 0 | 0 | 0 | 1 | 1 | 0 | 0 | 1 | 1 | 1 | 1 | 0 | 0 | 0 | 1 | 2 | 0 | 0 | 1 |
| 27 | *Paracaririneura* | 2 | 0 | 0 | 1 | 1 | 0 | 2 | 0 | 0 | 0 | 0 | 1 | 1 | 0 | 0 | 1 | 1 | 1 | 0 | 0 | 0 | 1 | 1 | 2 | 0 | 0 | 0 |
| 28 | *Araripeneura* | 2 | 0 | 0 | 1 | 1 | 0 | 2 | 0 | 0 | 0 | 0 | 1 | 1 | 0 | 0 | 1 | 1 | 1 | 1 | 0 | 0 | 1 | 1 | 2 | 0 | 0 | 0 |
| 29 | *Caldaida* | 2 | 0 | 0 | 1 | 1 | 0 | 2 | 0 | 0 | 0 | 0 | 1 | 1 | 0 | 0 | 1 | 1 | 1 | 1 | 0 | 0 | 0 | 1 | 2 | 0 | 0 | 0 |
| 30 | *Cratoalloneura* | 2 | 0 | 0 | 1 | 1 | 1 | 2 | 0 | 1 | 0 | 0 | 1 | 1 | 0 | 0 | 1 | 1 | 1 | 1 | 1 | 0 | 1 | 1 | 2 | 0 | 0 | 1 |
| 31 | *Allopteroneura* | 2 | 0 | 0 | 1 | 1 | 1 | 2 | 0 | 1 | 0 | 0 | 1 | ? | 0 | 0 | 1 | 1 | 1 | 1 | 1 | 0 | 1 | 1 | 2 | 0 | 0 | 1 |
| 32 | *Phylloleon* | 2 | 0 | 0 | 1 | 1 | 1 | 2 | 0 | 1 | 0 | 0 | 1 | 1 | 0 | 0 | 1 | 1 | 1 | 0 | 0 | 0 | 0 | 1 | 2 | 0 | 0 | 1 |
| 33 | *Burmaneura* | 2 | 0 | 0 | 1 | 1 | ? | 2 | 0 | 0 | 0 | 0 | ? | ? | 0 | 0 | 1 | ? | ? | ? | ? | 0 | ? | ? | 2 | 0 | ? | 0 |
| 34 | *Nanoleon* | 2 | 0 | 0 | 1 | 1 | 0 | 2 | 0 | 0 | 0 | 0 | 1 | 1 | 0 | 1 | 1 | 0 | 1 | 0 | 0 | 0 | 0 | 0 | 2 | 0 | 0 | 0 |
| 35 | *Bleyeria* | 2 | 0 | 0 | 1 | 1 | 0 | 2 | 0 | 0 | 0 | 0 | 1 | 1 | 0 | 0 | 1 | 0 | 0 | 0 | 0 | 0 | 0 | 0 | 2 | 0 | 0 | 1 |
| 36 | *Blittersdorffia* | 2 | 0 | 0 | 1 | 1 | 0 | 2 | 0 | 0 | 0 | 0 | 1 | 1 | 0 | 0 | 1 | 0 | 1 | 0 | 0 | 0 | 1 | 0 | 2 | 0 | 0 | 1 |
| 37 | *Pseudonymphes* | 2 | 0 | 0 | 0 | 1 | 0 | 2 | 0 | 0 | 0 | 0 | 1 | 1 | 0 | 0 | 1 | 0 | 1 | 0 | 0 | 0 | 0 | 0 | 0&2 | 0 | 0 | 1 |
| 38 | *Palpares* | 2 | 0 | 0 | 1 | 1 | 0 | 2 | 0 | 0 | 0 | 0 | 1 | 1 | 0 | 2 | 1 | 0 | 1 | 0 | 0 | 0 | 0 | 0 | 2 | 0 | 0 | 1 |
| 39 | *Stiobopteryx* | 2 | 0 | 0 | 1 | 1 | 0 | 2 | 0 | 0 | 0 | 0 | 1 | 1 | 0 | 2 | 1 | 0 | 1 | 0 | 0 | 0 | 0 | 0 | 2 | 0 | 0 | 1 |
| 40 | *Myrmeleon* | 2 | 0 | 0 | 1 | 1 | 0 | 2 | 0 | 0 | 0 | 0 | 1 | 1 | 0 | 2 | 1 | 0 | 1 | 0 | 0 | 0 | 0 | 0 | 2 | 0 | 0 | 1 |
| 41 | *Guyiling* | 1 | 0 | 0 | 1 | 1 | 0 | ? | 1 | 0 | 0 | 0 | 1 | ? | 0 | 0 | 0 | 0 | ? | 0 | 1 | 0 | 1 | ? | 2 | 0 | 0 | 1 |
| 42 | *Baisopardus* | 0 | 0 | 0 | 1 | 1 | 0 | 2 | 1 | 0 | 0 | 0 | 1 | 1 | 0 | 0 | 0 | 0 | 0 | 0 | 1 | 0 | 1 | 0 | 2 | 0 | 0 | 1 |
| 43 | *Parapalaeoleon* | 0 | 0 | 0 | 1 | 1 | 0 | 2 | 1 | 0 | 0 | 0 | 1 | 1 | 0 | 0 | 0 | 0 | 0 | 0 | 1 | 0 | 1 | 0 | 2 | 0 | 0 | 1 |
| 44 | *Paraneurastenyx* | ? | ? | 0 | 1 | 1 | 0 | 2 | 0 | 0 | 0 | 0 | 1 | 1 | 0 | 0 | 0 | 0 | 0 | 0 | 1 | 0 | 0 | ? | 2 | 0 | 0 | 1 |

|  |  | 28 | 29 | 30 | 31 | 32 | 33 | 34 | 35 | 36 | 37 | 38 | 39 | 40 | 41 | 42 | 43 | 44 | 45 | 46 | 47 | 48 | 49 | 50 | 51 | 52 | 53 | 54 |
| --- | --- | --- | --- | --- | --- | --- | --- | --- | --- | --- | --- | --- | --- | --- | --- | --- | --- | --- | --- | --- | --- | --- | --- | --- | --- | --- | --- | --- |
| 1 | *Gumilla* | 0 | 0 | 0 | 1 | 0 | 0 | 0 | 0 | 1 | 1 | 0 | 2 | 0 | 0 | 0 | 0 | 0 | 0 | 0 | 1 | 0 | 0 | 0 | 0 | 0 | 0 | 0 |
| 2 | *Ithone* | 1 | 0 | 0 | 0 | - | - | 0 | 0 | 1 | 0 | 0 | 1 | 0 | 0 | 0 | 0 | 0 | 0 | 0 | 0 | 0 | 0 | 0 | 1 | 0 | 0 | 0 |
| 3 | *Balmes* | 1 | 0 | 0 | 0 | - | - | 0 | 0 | 1 | 0 | 0 | 1 | 0 | 0 | 0 | 0 | 0 | 0 | 0 | 1 | 0 | 0 | 0 | 1 | 0 | 0 | 0 |
| 4 | *Nesydrion* | 0 | 0 | 0 | 0 | - | - | 0 | 0 | 1 | 0 | 0 | 0 | 0 | 0 | 0 | 0 | 0 | 0 | 2 | 1 | 0 | 0 | 1 | 1 | 0 | 0 | 0 |
| 5 | *Nymphes* | 1 | 1 | 1 | 0 | - | - | 0 | 0 | 1 | 0 | 0 | 0 | 0 | 0 | 0 | 0 | 0 | 0 | 0 | 1 | 0 | 0 | 1 | 1 | 0 | 0 | 0 |
| 6 | *Norfolius* | 0 | 0 | 0 | 0 | - | - | 0 | 0 | 2 | 0 | 0 | 2 | 0 | 0 | 0 | 0 | 0 | 0 | 0 | 1 | 0 | 0 | 1 | 1 | 0 | 0 | 0 |
| 7 | *Osmylops* | 0 | 0 | 0 | 0 | - | - | 0 | 0 | 2 | 0 | 0 | 2 | 0 | 0 | 0 | 0 | 0 | 0 | 0 | 1 | 0 | 0 | 1 | 1 | 0 | 0 | 0 |
| 8 | *Liminympha* | 1 | 0 | 0 | 0 | - | - | 0 | 0 | 2 | 1 | 0 | 2 | 0 | 0 | 0 | 0 | 0 | 0 | 0 | 1 | ? | ? | ? | ? | ? | ? | ? |
| 9 | *Cratosmylus* | ? | 1 | 0 | ? | ? | ? | 0 | 0 | 1 | ? | 1 | - | 0 | 0 | 0 | 0 | 0 | 1 | 2 | 1 | 1 | ? | ? | ? | ? | ? | ? |
| 10 | *Araripenymphes* | 2 | 1 | 0 | 0 | - | - | 0 | 1 | 0 | 0 | ? | ? | 0 | 0 | 0 | 0 | 0 | 1 | 2 | 1 | ? | ? | ? | ? | ? | ? | ? |
| 11 | *Neliana* | 1&2 | 1 | 0 | 0 | - | - | 0 | 1 | 1 | 1 | 0 | 0 | 0 | 0 | 0 | 0 | 0 | 2 | 2 | 1 | 1 | 0 | ? | ? | ? | ? | ? |
| 12 | *Parababinskaia* | 1&2 | 1 | 0 | 0 | - | - | 0 | 0 | 1 | 1 | 0 | 0 | 0 | 0 | 0 | 0 | 0 | 2 | 2 | 1 | 1 | 0 | 0 | 1 | ? | 0 | 0 |
| 13 | *Babinskaia* | 1 | 1 | 0 | 0 | - | - | 0 | 1 | 1 | 1 | 0 | 0 | 0 | 0 | 0 | 0 | 0 | 2 | 2 | 1 | 1 | 0 | ? | ? | ? | ? | ? |
| 14 | *Burmobabinskaia* | ? | 1 | 0 | 0 | - | - | 0 | 1 | 1 | 1 | 0 | 3 | ? | 2 | ? | 0 | 0 | 2 | 2 | 1 | 1 | 0 | 0 | 1 | ? | ? | ? |
| 15 | *Electrobabinskaia* | 2 | 1 | 0 | 0 | - | - | 0 | 1 | 1 | 1 | 0 | 3 | 1 | 0 | 0 | 0 | 0 | 2 | 2 | 1 | 1 | 0 | 0 | 1 | ? | 0 | 1 |
| 16 | *Pseudobabinskaia* | 1 | 1 | 0 | 0 | - | - | 0 | 1 | 1 | 1 | 0 | 3 | 0 | 0 | 0 | 0 | 0 | 2 | 2 | 1 | 1 | 0 | 0 | ? | ? | 0 | 1 |
| 17 | *Gigantobabinskaia* | ? | 1 | ? | 0 | - | - | 0 | 1 | 1 | 1 | 0 | 3 | 1 | 0 | 0 | 0 | 0 | 2 | 2 | 1 | 1 | 0 | 0 | ? | ? | ? | ? |
| 18 | *Calobabinskaia* | 1 | 1 | 0 | 1 | 0 | 0 | 0 | 0 | 3 | 0 | 1 | - | 0 | 0 | 0 | 0 | 0 | 2 | 2 | 1 | ? | 0 | 1 | ? | ? | ? | ? |
| 19 | *Stenobabinskaia* | 2 | 1 | 0 | 1 | 0 | 0 | 0 | 0 | 3 | 0 | 1 | - | 0 | 0 | 0 | 0 | 0 | 2 | 2 | 1 | 1 | 0 | ? | 1 | ? | ? | ? |
| 20 | *Xiaobabinskaia* | 2 | 1 | 0 | 0 | - | - | 0 | 0 | 1 | 1 | 1 | - | 1 | 0 | 0 | 0 | 0 | 2 | 2 | 1 | 1 | 0 | 0 | ? | ? | ? | 0 |
| 21 | *Roesleriana* | 2 | 1 | 1 | 0 | - | - | 1 | 0 | 1 | 0 | ? | ? | - | 2 | 2 | 0 | 0 | 0 | 0 | 1 | - | 0 | 0 | 1 | 0 | 0 | 0 |
| 22 | *Pastranaia* | 1 | 1 | 1 | 1 | 0 | 0 | 1 | 0 | 1 | 0 | 0 | 0 | - | 2 | 2 | 0 | 0 | 0 | 0 | 1 | - | 0 | 0 | 1 | 0 | 0 | 0 |
| 23 | *Choromyrmeleon* | 1 | 1 | 1 | 1 | 1 | 0 | 0 | 0 | 1 | 0 | 0 | 3 | 0 | 0 | 0 | 0 | 0 | 1 | 0 | 1 | ? | ? | ? | ? | ? | ? | ? |
| 24 | *Caririneura* | 1 | 1 | 1 | 1 | 0 | 0 | 1 | 1 | 1 | 0 | 0 | 2 | 1 | 1 | 0 | 0 | 0 | 2 | 2 | 1 | ? | ? | ? | ? | ? | ? | ? |
| 25 | *Cratoneura* | 2 | 1 | 1 | 0 | - | - | 1 | 1 | 1 | 0 | ? | ? | 1 | 0 | 0 | 1 | 1 | 1 | 0 | 1 | ? | ? | ? | ? | ? | ? | ? |
| 26 | *Cratopteryx* | 1 | 1 | 1 | 1 | 0 | 0 | 1 | 0 | 1 | 0 | ? | ? | 1 | 0 | 0 | 0 | 0 | 1 | 0 | 1 | ? | ? | ? | ? | ? | ? | ? |
| 27 | *Paracaririneura* | 1 | 1 | 1 | 1 | 0 | 0 | 1 | 0 | 1 | 0 | 0 | 3 | ? | ? | ? | ? | ? | ? | ? | ? | ? | ? | ? | ? | ? | ? | ? |
| 28 | *Araripeneura* | 1 | 1 | 1 | 1 | 0 | 0 | 1 | 1 | 1 | 0 | 0 | 3 | 1 | 0 | 0 | 0 | 0 | 1 | 0 | 1 | ? | ? | ? | ? | ? | ? | ? |
| 29 | *Caldaida* | ? | ? | ? | ? | ? | ? | 1 | 1 | ? | ? | ? | ? | 1 | 0 | 0 | 1 | 1 | ? | ? | ? | ? | ? | ? | ? | ? | ? | ? |
| 30 | *Cratoalloneura* | 1 | 1 | 1 | 1 | 0 | 0 | 1 | 1 | 1 | 0 | 0 | 0 | 1 | 0 | 0 | 1 | 1 | 1 | 0 | 1 | ? | ? | ? | ? | ? | ? | ? |
| 31 | *Allopteroneura* | 2 | 1 | 1 | 1 | 0 | 0 | 1 | 1 | 1 | 0 | 0 | 0 | 1 | 0 | 0 | 1 | 1 | 1 | 0 | 1 | ? | 1 | 0 | ? | ? | ? | ? |
| 32 | *Phylloleon* | 1&2 | 1 | 1 | 1 | 0 | 0 | 1 | 0 | 1 | 0 | 0 | 0 | 1 | 0&1 | 0&1 | 1 | 1 | 2 | 2 | 1 | 0 | 1 | 0 | ? | ? | 1 | 0 |
| 33 | *Burmaneura* | 1 | 1 | 1 | 0 | - | - | 0 | 0 | 0 | 0 | 0 | 3 | 0 | 0 | 0 | 0 | 0 | 1 | 1 | 1 | 0 | 1 | ? | ? | ? | ? | ? |
| 34 | *Nanoleon* | 1 | 1 | 1 | 1 | 1 | 0 | 0 | 0 | 0 | 0 | 0 | 3 | 0 | 0 | 0 | 0 | 0 | 1 | 1 | 1 | 0 | 1 | 0 | ? | ? | 1 | 0 |
| 35 | *Bleyeria* | 1 | 1 | 1 | 1 | 1 | 1 | 0 | 0 | 0 | 0 | 0 | 0 | 0 | 0 | 0 | 0 | 0 | 1 | 0 | 1 | ? | ? | ? | ? | ? | ? | ? |
| 36 | *Blittersdorffia* | 1&2 | 1 | 1 | 1 | 1 | 1 | 0 | 0 | 0 | 0 | 0 | 0 | 0 | 0 | 0 | 0 | 0 | 1 | 0 | 1 | ? | ? | ? | ? | ? | ? | ? |
| 37 | *Pseudonymphes* | 0&1 | 1 | 1 | 1 | 1 | 1 | 0 | 0 | 0 | 0 | 0 | 2 | 0 | 0 | 0 | 0 | 0 | 1 | 0 | 1 | 0 | ? | ? | ? | ? | ? | ? |
| 38 | *Palpares* | 2 | 1 | 1 | 1 | 1 | 0 | 1 | 1 | 0 | 0 | 0 | 0 | 0 | 0 | 0 | 0 | 0 | 1 | 2 | 1 | 0 | 1 | 0 | 1 | 1 | 1 | 0 |
| 39 | *Stiobopteryx* | 2 | 1 | 1 | 1 | 1 | 0 | 1 | 0 | 0 | 0 | 0 | 0 | 0 | 0 | 0 | 0 | 0 | 1 | 1 | 1 | 0 | 1 | 0 | 1 | 1 | 1 | 0 |
| 40 | *Myrmeleon* | 2 | 1 | 1 | 1 | 1 | 0 | 1 | 1 | 0 | 0 | 0 | 0 | 0 | 0 | 0 | 0 | 0 | 1 | 2 | 1 | 0 | 1 | 0 | 1 | 1 | 1 | 0 |
| 41 | *Guyiling* | 2 | 1 | 1 | 1 | 1 | 1 | 0 | 0 | 0 | 0 | ? | ? | ? | ? | 0 | 0 | 0 | 0 | 0 | 1 | ? | 0 | ? | ? | ? | ? | ? |
| 42 | *Baisopardus* | 2 | 1 | 1 | 1 | 1 | 2 | 0 | 0 | 0 | 0 | 0 | 3 | 0 | 0 | 0 | 0 | 0 | 0 | 0 | 1 | 0 | 0 | ? | ? | ? | ? | ? |
| 43 | *Parapalaeoleon* | 2 | 1 | 1 | 1 | 1 | 2 | 0 | 0 | 0 | 0 | 0 | 3 | 0 | 0 | ? | ? | ? | ? | ? | ? | ? | ? | ? | ? | ? | ? | ? |
| 44 | *Paraneurastenyx* | 2 | 1 | 1 | 1 | 1 | 1 | 0 | 0 | 0 | 0 | 0 | 0 | ? | ? | ? | ? | ? | ? | ? | ? | ? | ? | ? | ? | ? | ? | ? |

**Supplementary references** (number continued with that in References in the main text)

57. Wang, X.-l. & Bao, R. A taxonomic study on the genus *Balmes* Navás from China (Neuroptera, Psychopsidae). *Acta Zootaxon. Sin.* **31**, 846–850 (2006).

58. Riek, E. F. The Australian moth-lacewings (Neuroptera: Ithonidae). *J. Aust. Entomol. Soc.* **13**, 37–54 (1974).

59. Califre Martins, C., Ardila-Camacho, A. & Aspöck, U. Neotropical osmylids (Neuroptera, Osmylidae): three new species of *Isostenosmylus* Krüger, 1913, new distributional records, redescriptions, checklist and key for the Neotropical species. *Zootaxa* **4149**, 1–66 (2006).
